# The International Trade of Ware Vegetables and Orna-Mental Plants—An Underestimated Risk of Accelerated Spreading of Phytopathogenic Bacteria in the Era of Globalisation and Ongoing Climatic Changes

**DOI:** 10.3390/pathogens11070728

**Published:** 2022-06-26

**Authors:** Magdalena Smoktunowicz, Joanna Jonca, Aneta Stachowska, Michal May, Michal Mateusz Waleron, Malgorzata Waleron, Krzysztof Waleron

**Affiliations:** 1 Department of Pharmaceutical Microbiology, Faculty of Pharmacy, Medical University of Gdansk, 80-416 Gdansk, Poland; magdalena.smoktunowicz@gumed.edu.pl; 2 Laboratory of Plant Protection and Biotechnology, Intercollegiate Faculty of Biotechnology of the University of Gdansk and the Medical University of Gdansk, 80-307 Gdansk, Poland; joanna.jonca@ug.edu.pl (J.J.); aneta.stachowska@gumed.edu.pl (A.S.); michal.may@phdstud.ug.edu.pl (M.M.); michal.waleron@gumed.edu.pl (M.M.W.)

**Keywords:** *recA*, *Pectobacterium*, *Dickeya*, adaptive potential, bacterial spread, pathogenicity, antibiotic resistance

## Abstract

Bacteria of the genus *Pectobacterium* are globally occurring pathogens that infect a broad spectrum of plants. The plant cell wall degrading enzymes allow them to cause diseases like soft rot and blackleg. Worldwide trade and exchange of plant material together with the accompanying microorganisms contributed to the rapid spread and consequently the acquisition of new traits by bacteria. The 161 pectinolytic strains were isolated from symptomless vegetables and ornamental plants acquired from Polish and foreign local food markets. All strains except four *Dickeya* isolates were identified as belonging to the *Pectobacterium* genus by PCR with species-specific primers and *recA* gene sequencing. The newly isolated bacteria were assigned to eight species, *P. versatile* (50 strains), *P. carotovorum* (33), *P. brasiliense* (27), *P. atrosepticum* (19), *P. parmentieri* (12), *P. polaris* (11), *P. parvum* (3) and *P. odoriferum* (2). ERIC PCR and phenotypic characteristics revealed high heterogeneity among *P. carotovorum*, *P. brasiliense* and *P. versatile* isolates. Moreover, a subset of the newly isolated strains was characterised by high tolerance to changing environmental conditions such as salinity, pH and water availability. These bacteria can effectively macerate the tissues of various plants, including potato, chicory and orchid. Our results indicate that *Pectobacterium* strains isolated from internationally traded, symptomless vegetables and ornamental plants have high potential for adaptation to adverse environmental conditions and to infect various host plants. These features may contribute to the success of the genus *Pectobacterium* in spreading between different climatic zones and facilitate the colonisation of different ecological niches.

## 1. Introduction

Bacteria of the genus *Pectobacterium* are widespread and cause many diseases on a wide range of economically important plants worldwide. Those bacteria have a broad host range, and most do not demonstrate specificity for host plants. Although the genus *Pectobacterium* is known mainly as plant pathogens, they may also be saprophytes [1]. Nevertheless, the infections with these pathogens lead to severe economic losses of horticultural and ornamental plants in the fields and during their storage. Thus, the genus is recognised as one of the ten most important bacterial plant pathogens and is the subject of research by numerous groups of scientists [2].

The taxonomy of the genus *Pectobacterium* is being constantly rearranged. Currently, twenty different *Pectobacterium* species, *P. actinidiae*, *P. aquaticum*, *P. aroidearum*, *P. atrosepticum*, *P. betavasculorum*, *P. brasiliense*, *P. cacticidum*, *P. carotovorum*, *P. fontis*, *P. odoriferum*, *P. peruviense*, *P. polaris*, *P. polonicum*, *P. parmentieri*, *P. parvum*, *P. punjabense*, *P. quasiaquaticum*, *P. versatile*, *P. wasabiae* and *P. zantedeschiae* have been described [3]. 

Most *Pectobacterium* species can inhabit and infect different plant species; however, *P. aroidearum* and *P. zantedeschiae* have been mainly isolated from monocotyledonous [4,5]. Strains of *P. wasabiae* are isolated from horseradish in Japan [6], *P. actinidiae* from kiwi [7], and *P. betavasculorum* derived from sugar beets, potato, artichoke, and sunflower [8]. Apart from this, *P. cacticidum* occurs only in desert regions where bacteria inhabit different cacti [9], while the species *P. atrosepticum* is much more common in cooler climates, and it is isolated from potatoes most often [10]. It is likewise for recently described species *P. polaris* [11], *P. parvum* [12], *P. parmentieri* [13], *P. peruviense* [14], and *P. punjabense* [15,16]. 

However, the species *P. carotovorum*, *P. brasiliense*, *P. odoriferum* and *P. versatile* have been isolated from various plants and different climatic zones [3,17,18]. Moreover, *P. versatile* [19], *P. odoriferum* [20] and *P. brasiliense* [21] have been isolated from invertebrates. In contrast, *P.* *fontis* [22], *P. aquaticum* [23], *P. polonicum* [24] and *P. quasiaquaticum* [25] have been isolated from water.

It can be noted that the cardinal temperatures for individual *Pectobacterium* species [26] correlate with climatic regions in which the common occurrence of particular species is recorded. For example, the growth temperature for the *Pectobacterium* genus ranges from 20 °C to 34 °C and 43 °C for *P. cacticidum* [9,26]. 

In addition to the differences in the optimal growth temperature, species within the genus *Pectobacterium* are biochemically diverse. They differ in the use of nutrients and the production of plant cell wall degrading enzymes (PCWDEs) like pectin lyases, cellulases, proteases or amylases [27]. However, the analysis of physiological and biochemical properties of *Pectobacterium* strains does not provide sufficient information to classify newly isolated strains to a particular species [28]. Detailed identification of *Pectobacterium* strains is possible by PCR that uses primers specific for a given species, e.g., *P. atrosepticum* [29], *P. brasiliense* [30] or *P. wasabiae* and *P. parmentieri* [31]. Unfortunately, the primers developed so far allow for effective detection of *P. atrosepticum* only. The primers proposed by Darrasse et al. [32] enable the detection of bacteria from the genus *Pectobacterium*, except for the species *P. betavasculorum.* Likewise, primers proposed for detecting *P. parmentieri* [31] and *P. brasiliense* [30] do not seem to be sufficiently universal. Currently, the accurate identification of *Pectobacterium* species relies on gene sequencing methods. The Multi Locus Sequence Analysis (MLSA) is the most effective method to identify *Pectobacterium* species [14,19,33]. While analysing the genetic diversity of bacteria of the genus *Pectobacterium*, the rep-PCR reaction with ERIC primers [34] has been successfully applied [5,14].

The aim of this study was to check whether the ware vegetables or ornamental plants that can be purchased in the market can transmit bacteria from the genus *Pectobacterium.* The sequencing of the *recA* gene was applied to identify newly isolated *Pectobacterium* strains. The comprehensive characteristics of phenotypic features, such as biochemical properties, ability to macerate different plant species and tolerance to different pH, temperatures, salinity, or water availability, were performed. This phenotypic biology allows us to determine whether the isolated strains have the potential for adaptation to adverse environmental conditions and successful spreading between different climatic regions.

## 2. Results

### 2.1. Species Identification and Diversity Analysis

We collected 241 samples of asymptomatic ware vegetable and ornamental plant samples (110 of which were potato), among which 149 (62%) were carrying pectinolytic bacteria (65 of which were potato, 43 vegetables and 41 ornamental and herbaceous). The number of pectinolytic bacteria detected in the tested samples differed. The highest number of pectinolytic bacteria, 10^4^ of bacterial cells on surface equal to 1 cm^2^ of the sample was observed for unwashed vegetables stuck with soil. For most of the samples from which we isolated pectinolytic bacteria, their estimated numbers ranged from 10^2^ to 10^3^ per 1 cm^2^ of plant sample.

The initial species identification was carried out by PCR, using primers specific for *Pectobacterium* and *Dickeya* genera [32,35]. Among 161 pectinolytic strains, 157 could be classified into the *Pectobacterium* and four into the *Dickeya* genus (Table 1). 

Apart from the 161 isolates mentioned above, another five isolates, which did not belong to the genus *Pectobacterium* or *Dickeya* were isolated. They were identified by *recA* gene sequencing as belonging to *Serratia* spp. (strains DPMP88, DPMP93 and DPMP337), *Klebsiella oxytoca* (DPMP79), and *Rahnella aquatilis* (DPMP382). These strains of *Serratia*, *Klebsiella* and *Rahnella* were not included in further analyses.

Furthermore, among 157 strains identified as *Pectobacterium*, nineteen strains were classified as *P. atrosepticum*, twelve strains isolated from symptomless potatoes as *P. parmentieri*, and ten strains were identified as *P. brasiliense*, based on species-specific PCR reactions [29,30,31]. Unfortunately, 115 out of 157 newly isolated strains could not be classified to the species level with applied species-specific primers (Table 1).

The genetic diversity of 161 new *Pectobacterium* and *Dickeya* isolates originating from symptomless plants and 31 reference strains isolated from symptomatic plants was assessed by the ERIC-PCR method. In total, 23 different ERIC profiles were discriminated (Table 1).

In the next step, based on obtained results, 75 strains with different ERIC fingerprints and different geographical origins or isolated from distinct plant materials were selected for the *recA* gene sequencing. Finally, the obtained sequences of newly isolated strains were compared with the *recA* gene sequences available in the Genbank and the phylogenetic analysis was conducted. 

**Figure 1:**
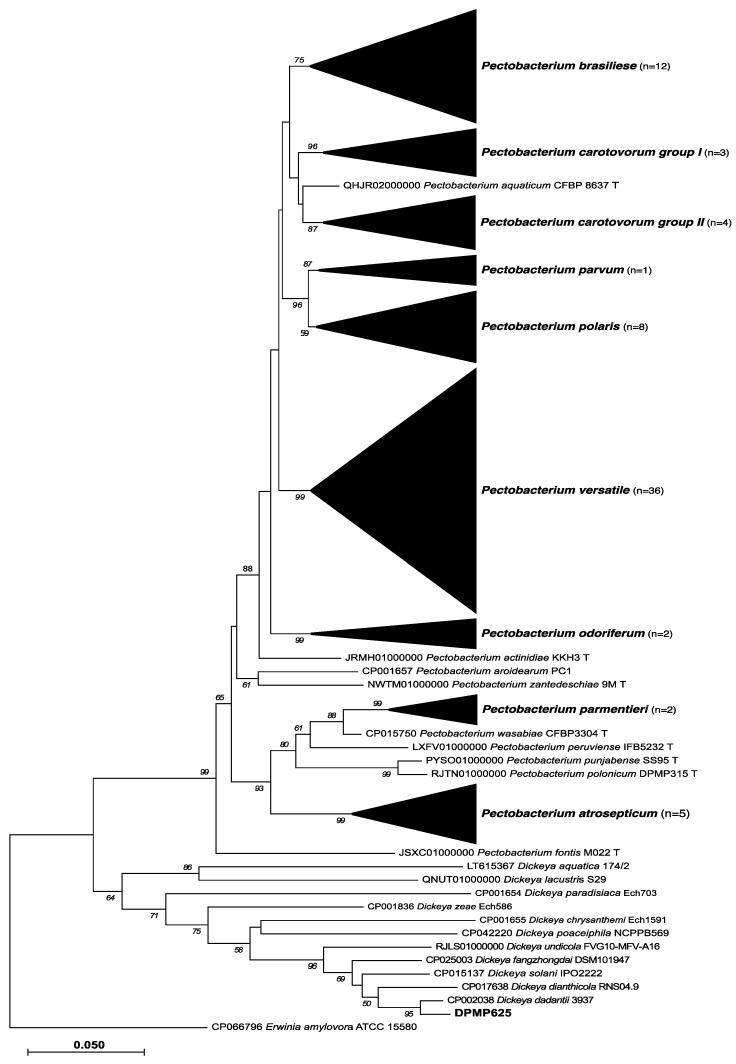
The *recA* gene sequences-based phylogeny of *Pectobacterium* and *Dickeya* strains isolated from asymptomatic plants. The maximum likelihood tree based on *recA* gene sequences reflecting the phylogenetic position of 73 newly isolated *Pectobacterium* and 1 *Dickeya* strains originated from asymptomatic plant samples and 18 strains originated from symptomatic plants. For comparison, the 44 sequences of type strains and reference strains from both genera were retrieved from Genbank and were included in the analysis. The number in the brackets indicates the number of newly isolated strains present in each clade. The bootstrap value was equal to 1000 replicates. The *recA* gene sequence of *Erwinia amylovora* was used as an outgroup. Bootstrapping values < 50% were cut off.

The topology of the maximum likelihood tree based on the *recA* gene sequences clearly shows the phylogenetic position of newly isolated *Pectobacterium* and *Dickeya* strains. The *recA* gene sequences of 73 *Pectobacterium* and 1 *Dickeya* strains that have been isolated from asymptomatic plants were grouped together with 26 strains originated from symptomatic plants, as well as with *Pectobacterium* spp. and *Dickeya dadantii* type and reference strains (Figure 1).

**Figure 2:**
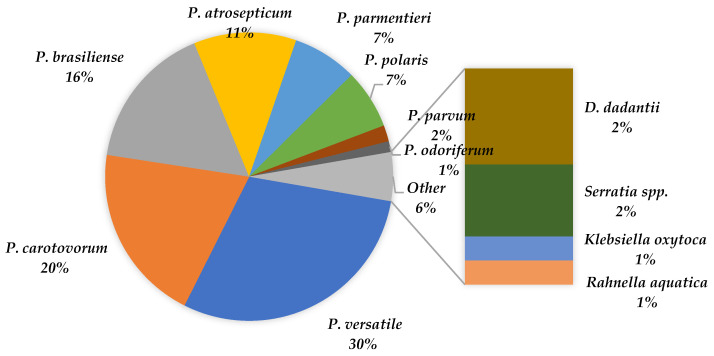
Percentage share of individual *Pectobacterium* species and other genera of pectinolytic bacteria detected in the tested samples of vegetables and ornamental plants that did not show any disease symptoms.

Out of the 160 pectinolytic strains, 157 isolates (98%) were identified as *Pectobacterium* and classified into the following species: *P. versatile* (50 isolates), *P. carotovorum* (33 isolates), *P. brasiliense* (27 isolates), *P. atrosepticum* (19 isolates), *P. parmentieri* (12 isolates), *P. polaris* (11 isolates), *P. parvum* (3 isolates), and *P. odoriferum* (2 isolates) (Figure 2, Table 1).

The most abundant species were *P. versatile* (30%), *P. carotovorum* (20%), *P. brasiliense* (16%), and *P. atrosepticum* (11%) that have been isolated from different plant species (vegetables and ornamental plants as well), originating from different countries. Only from Potatoes have we isolated all detected *Pectobacterium* species (Table 1). 

It should be noted that different *Pectobacterium* species can occur on the same asymptomatic plant sample. For example, the presence of two species, *P. brasiliense* and *P. polaris*, has been detected on Sugar beet sample 111 (DPMP394 and DPMP403), Bittersweet sample 112 (DPMP396 and DPMP397) and Moroccan potato tuber sample 13 (DPMP55 and DPMP730). On the other hand, from the same potato tuber samples, the other combinations of *Pectobacterium* species have been isolated. *P. atrosepticum* and *P. versatile* were detected in Potato stem sample 98 from Poland (DPMP344 and DPMP133) and Potato tuber from Belgium (DPMP133 and DPMP134). *P. atrosepticum* DPMP278 was co-isolated with *P. polaris* DPMP280 from Potato tuber sample 73 from Norway. Meanwhile, in Poland, the *P. versatile* DPMP352 strain was co-isolated with the *P. parmentieri* DPMP353 strain from the same sample of potato tuber 99.1. Another two *Pectobacterium* species combinations, *P. parmentieri* DPMP370 and *P. carotovorum* DPMP369 and *P. parvum* DPMP20 and *P. versatile* MKW18 were extracted from Cyprus potato tuber samples number 100 and mkw18, respectively. Furthermore, three different species: *P. brasilense* (strain DPMP152), *P. carotovorum* (strain DPMP146), and *P. versatile* (strain DPMP155) were observed on the same potato tuber sample number 44 from Morocco. Meanwhile, from Potato tuber sample number 155 from Kazakhstan, *P. atrosepticum* DPMP634, *P. carotovorum* DPMP331 and *P. versatile* DPMP632 and DPMP633 were detected (Table 2).

### 2.2. Phenotypic Characteristics 

#### 2.2.1. Plant Tissue Maceration 

**Figure 3:**
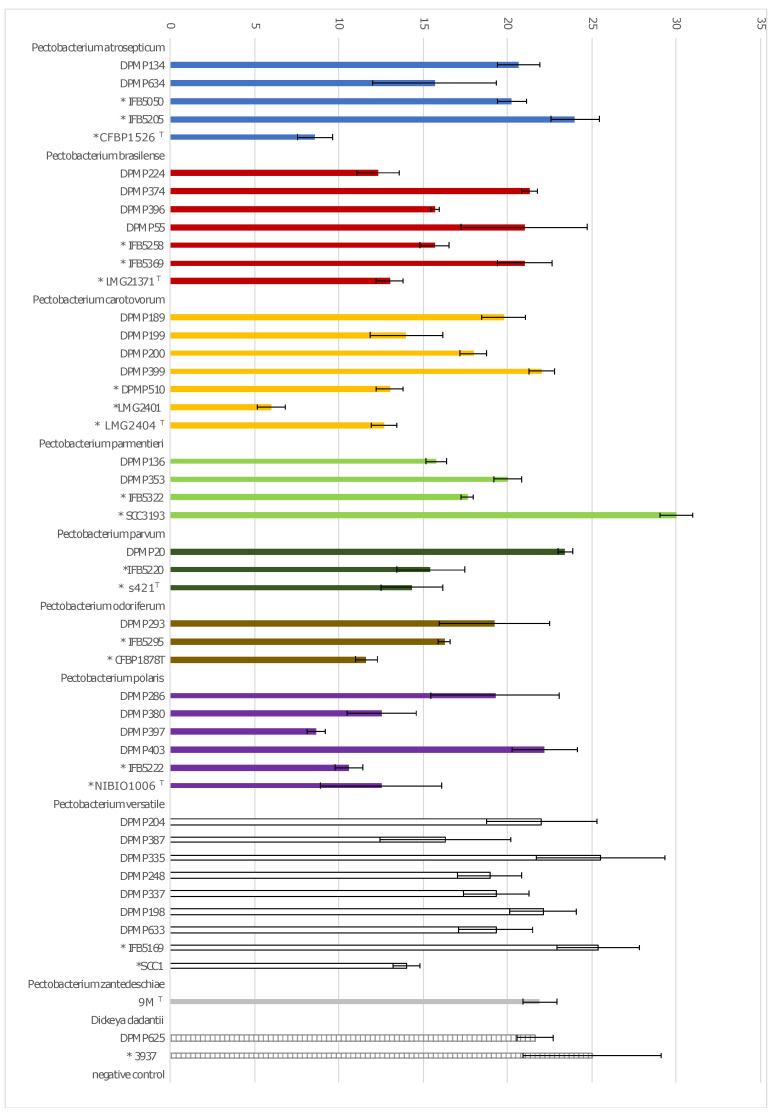
Comparison of the potato tissue maceration ability of the 26 strains isolated from asymptomatic plant samples in contrast to 21 strains originating from plants with disease symptoms. Strains abbreviations: * strains isolated from symptomatic plants, ^T^—type strains. Means ± SD of diameters of the rotten tissues is depicted. Three independent experiments with three replications were conducted.

**Figure 4:**
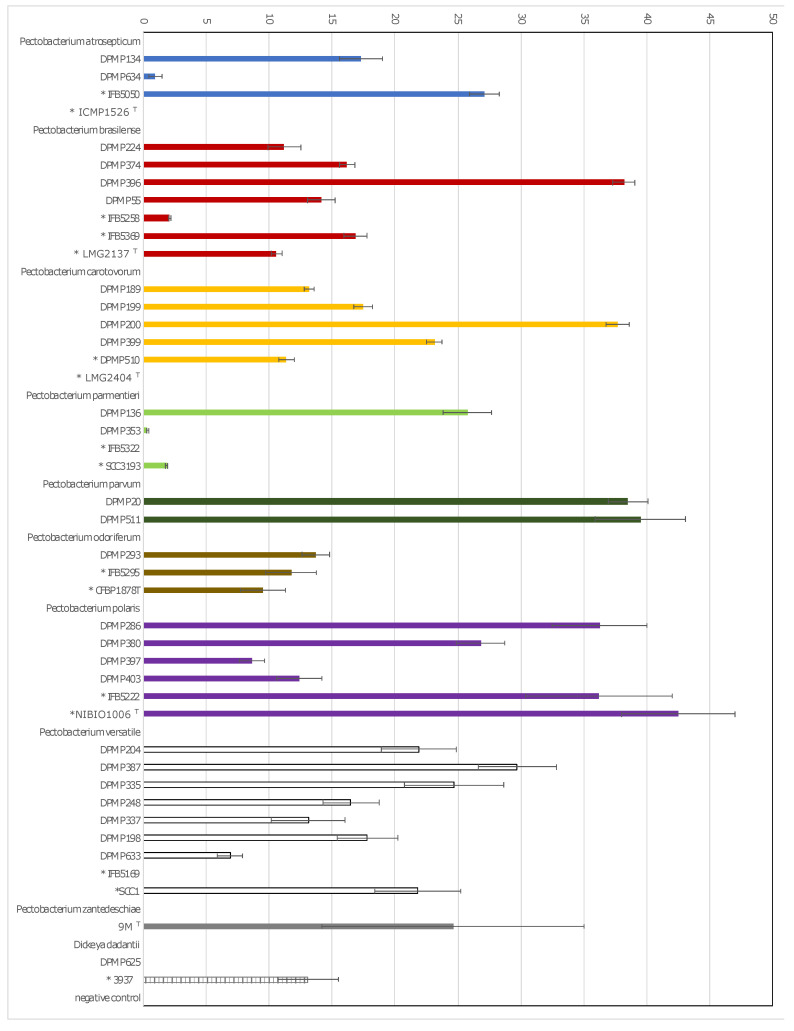
Comparison of the chicory tissue maceration ability of the 26 strains isolated from asymptomatic plant samples in contrast to 18 strains originating from plants with disease symptoms. Strain abbreviations: * strains isolated from symptomatic plants, ^T^—type strains. Means ± SD of diameters of the rotten tissues is depicted. Three independent experiments with three replications were conducted.

The ability to macerate plant tissue was investigated for randomly selected strains representing each of the isolated eight *Pectobacterium* and one *Dickeya* species. The number of strains selected for adaptation tests reflects the percentage of strains classified into each *Pectobacterium* species detected on asymptomatic plants. The pathogenicity of 47 strains was assessed by maceration of potato tuber, while, for 44 strains, the assay was performed on chicory and iris leaves. None of the selected strains caused disease on Iris leaves (data not shown). All the tested strains were capable to macerate potato tuber tissues; among them, 41 strains caused soft rot of potato tubers and chicory leaves also. However, five strains: *P. atrosepticum* DPMP634, *P. brasiliense* IFB5258, *P. parmentieri* DPMP353 and SCC3193, as well as *P. versatile* DPMP633 macerated efficiently potato tubers but exhibited weak pathogenicity on chicory. Other four strains, *P. atrosepticum* ICMP1526^T^, *P. carotovorum* LMG2404^T^, *P. parmentieri* IFB5322, and *D. dadantii* DPMP625, caused maceration of potato tuber tissue and did not cause significant damage to chicory leaves (Figure 3 and Figure 4, Tables S1 and S2). 

Of 47 strains for which the potato tuber maceration capacity was tested, 27 were isolated in this study from asymptomatic plant samples, and the remaining 20 (marked with asterisks in Table S1 and Figure 3 and Figure 4) were the reference strains or originated from plants with disease symptoms. Both groups of strains were characterised by the ability to macerate both potato tubers and chicory leaves.

**Figure 5:**
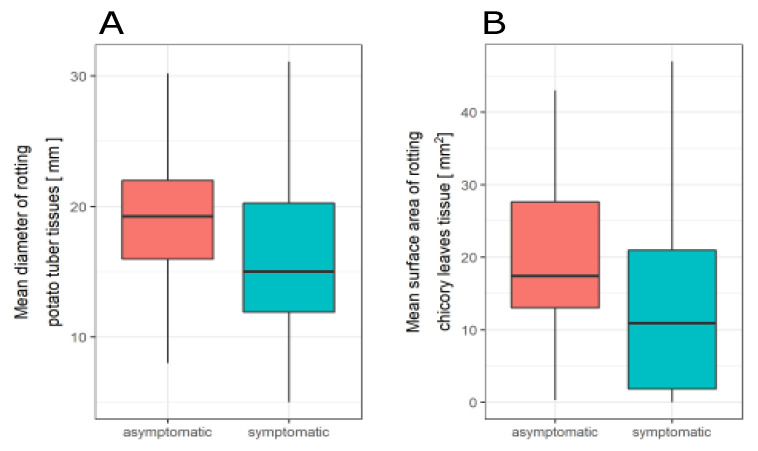
Comparison of the (**A**) potato tuber and (**B**) chicory leaves tissue maceration ability of the strains isolated from asymptomatic and symptomatic plant samples. *p* = 0.02.

Strains isolated from symptomless plants have nearly similar ability to macerate plant tissues as strains retrieved from plants with disease symptoms (Figure 3 and Figure 4, Figures S1 and S2, Tables S1 and S2). However, for the strains originated from symptomatic plants, a slightly smaller rot area was observed than for asymptomatic strains (Figure 5, Table S2). The *p*-value was 0.02 according to the ANOVA with Welch corrections for nonhomogeneous variances criterion followed by a post-hoc Games–Howell analysis (Figure 5). 

#### 2.2.2. Adaptation for Various Environmental Conditions

Adaptation tests for different environmental conditions: variable pH, salinity levels and water availability were performed for 35 *Pectobacterium* strains (Table 3, Table 4 and Table 5). Strains representing each of identified *Pectobacterium* species were randomly selected. 

##### pH Influence on Bacterial Growth

All tested strains showed the ability to grow in a Tryptic Soy Broth (TSB) medium with a pH of 5 to 10 but their growth efficiency declined with the more alkaline pH. Only five strains, one strain *P. brasiliense* DPMP55, isolated from a Moroccan potato, and two strains of *P. carotovorum* DPMP199 and DPMP200, also isolated from potatoes originating in Egypt, as well as *P. polaris* DPMP286 and *P. versatile* DPMP198 were able to tolerate low pH (pH= 4). On the contrary, the highest pH value = 11 was tolerated by all strains except two *P. brasiliense* strains, DPMP68 from symptomatic potato and DPMP396 from Bittersweet, and one *P. polaris* DPMP403 from Sugar beet grown in Poland. All tested strains showed their optimum growth at a slightly acidic pH of 5 or 6, except that *P. brasiliense* strain DPMP396 and *P. parvum* DPMP20, which showed their optimal at a neutral pH = 7. Only three strains, *P. brasiliense* DPMP55, *P. polaris* DPMP286, and *P. versatile* DPMP198 were able to grow and were metabolically active in all tested pH ranges, from 4 up to 11 (Table 3).

##### The Salinity Impact on Bacterial Growth

Most of the tested strains (31 out of 35) showed their optimum growth at very low salinity up to 1% of NaCl in the medium. Only two strains, *P. brasiliense* DPMP396, and *P. carotovorum* DPMP199 grew best in medium containing 4 and 3% NaCl, respectively. Three *P. parmentieri* strains DPMP390, IFB5322, and SCC3193 were the most salinity-sensitive and only grew in a medium containing less than 4% NaCl. A vast spectrum of salinity from 0 to 8% NaCl in the medium was tolerated by the strain *P. brasiliense* DPMP55. In salinity equal to 11%, none of the tested isolates maintained an active metabolism, as evidenced by the resazurin test. However, ten strains, *P. carotovorum* DPMP199 and DPMP399, *P. brasiliense* DPMP55, DPMP224, DPMP372, DPMP396, and DPMP374, *P. atrosepticum* DPMP275, *P. versatile* DPMP402 and DPMP452 can survive in medium containing 11% of NaCl and were viable in a spot test performed on an MH medium without NaCl (Table 4).

##### The Impact of Variable Water Availability on Bacterial Growth

All tested strains achieved the highest optical density value in medium without the addition of PEG. Out of 35 tested isolates, 13 were viable at PEG concentration above 200 g L^−^^1^. Four *Pectobacterium* strains were viable in the TSB medium containing 300–400 g L^−1^ PEG, and two *P. versatile* strains DPMP198 and DPMP202 were isolated from cactus and *P. carotovorum* strains DPMP199 and DPMP200 that were isolated from potato that was grown in Egypt. The most resistant was one *P. carotovorum* strain DPMP202 isolated from cactus, which remained viable and active metabolically at a concentration of PEG equal to 500 g L^−1^ (Table 5).

#### 2.2.3. Antibiotic Susceptibility Test and Growth on Chromogenic Media for Antibiotic Resistance Detection

Due to the lack of EUCAST guidelines for *Pectobacterium* spp. strains, it was assumed that the zone of growth inhibition in the range of 0–6 mm means antibiotic-resistant strains, growth inhibition zone in the range of 7–13 mm means moderately sensitive strains for a given antibiotic, and a zone of growth inhibition >13 mm means strains sensitive to a given antibiotic.

Among 34 tested isolates, 13 strains, were resistant to ampicillin, 9 strains to erythromycin, 2 to gentamicin, 6 strains to kanamycin, and 5 to streptomycin and tetracycline (Table 6).

The most susceptible to tested antibiotics were *P. atrosepticum*, *P. brasiliense* and *P. polaris* strains. The latter one revealed sensitivity to all of tested antibiotics. In contrast, *P. versatile* strains were most resistant among tested *Pectobacterium* species. Seven out of 9 *P. versatile* strains were resistant to ampicillin, 5 to the erythromycin, 4 to kanamycin and tetracycline, 3 to streptomycin, and 2 were growing in the presence of gentamicin. 

We additionally observed that some strains of *P. brasiliense*, *P. carotovorum* and *P versatile* were able to grow on ESBL, while 3 *P. versatile*, 1 *P. odoriferum* and 1 *P. parmentieri* strains grew on CARBA chromagar plates (Table 6).

Two *P. versatile* strains DPMP198 and DPMP202, which were isolated from cactus and strain DPMP633 from Kazakhstan, were resistant to all tested antibiotics and revealed an ESBL type of resistance. Three other *P. versatile* strains DPMP108 and DPMP112 isolated from Iris and DPMP181 from zucchini in Poland exhibited resistance to carbapenems. 

## 3. Discussion

In the period of 1999–2018, we collected 241 samples of asymptomatic ware vegetable, ornamental, and herbaceous plant samples. From 149 (62%) of symptomless plants that came from twenty-two countries from Africa, America, Asia, and Europe samples, we have isolated 161 pectinolytic bacteria. 

Genetic identification revealed that 98% of isolates belonged to eight *Pectobacterium* spp., and only 2% of isolates were classified as *Dickeya*. However, it should be noted that bacteria were isolated at temperature 28 °C, which is not optimal for *Dickeya* growth. 

Among *Pectobacterium* species, *P. versatile* was most frequently isolated from symptomless plants. Likewise, it was the most abundant *Pectobacterium* species deposited in CIRM-CFBP—a French collection of plant pathogenic bacteria [36]. Furthermore, this observation agrees with our earlier studies from 2001 and 2002 [37,38]. Analysis of the *recA* PCR-RFLP profiles for strains present in Polish and international collections of *Erwinia carotovora* indicated that only seven profiles: 3, 4, 5, 6, 7, 13 and 18 were common for both groups. Four profiles: 3, 4, 5, 6 were predominant in Polish and worldwide collection, and, in both populations, about 44% of the collected strains belonged to profile number 4. Currently, profiles 4 and 5 gather *P. versatile* strains, while profiles 3 and 6 are characteristic for *P. parmentieri* and *P. polaris* species, respectively. The sequencing of the *recA* gene for strains that have been used for RFLP analysis of an amplified fragment of *recA* gene confirmed that the most frequently observed RFLP profiles 4 and 5 are typical for *P. versatile* species. 

Furthermore, *P. versatile* was also most frequently isolated from water [39]. Thus, it is possible to conclude that, for the last twenty years, *P. versatile* continues to be the most frequently isolated taxon among the *Pectobacterium* species.

Strains belonging into *P. versatile* together with *P. brasiliense*, *P. carotovorum*, and *P. polaris* are the most divergent among *Pectobacterium* species. For each species, four different fingerprinting profiles were observed. In the case of *P. atrosepticum* and *P. parmentieri*, three and two ERIC profiles were determined, respectively. The above observations are in line with numerous reports on genetic diversity within the species belonging to the genus *Pectobacterium.* It should be noted that strains isolated from symptomatic plants do not differ genetically from those isolated from asymptomatic plants. Both groups of strains are assigned to the same fingerprinting profiles and *recA* gene sequences. 

It should be emphasised that, in this study, we have detected the presence of the same *Pectobacterium* and *Dickeya* species on asymptomatic vegetables and ornamental plants as those which occurred on plants with disease symptoms in the same countries from which we have analysed samples (Brasil, Egypt, Morocco, UK, USA, Israel, Finland, Norway, the Netherlands and Poland) [3,12,17,18,21,40,41,42,43,44,45,46,47,48,49,50,51]. In addition, the presence of virulent strains of the same species of *Pectobacterium* and *Dickeya* has been described in the rivers of Finland [45] and France [39].

We also observed that different *Pectobacterium* species can occur on the same asymptomatic plant sample. So far, the co-occurrence of various *Pectobacterium* species on the same plant samples have been noted on the symptomatic plants only [3]. Based on the obtained results, we can conclude that in the same sample of the plant we did not observe the coexistence of *P. brasiliense* with *P. atrosepticum* or *P. parmentieri.* It can be assumed that the strains belonging to the above-mentioned species may be antagonistic towards each other. It has been experimentally shown that *P. brasiliense* PBR1692^T^ produces bacteriocins against *P. atrosepticum* SCRI1043 and *P.* *carotovorum* WPP14 [52]. Indeed, in our observations, we did not observe strains of *P. brasilense* co-existing on the same plant sample with *P. atrosepticum* or *P. parmentieri* strains. Besides bacteriocins, some strains of *Pectobacterium*, *Dickeya* and *Serratia* (all these species were detected by us on asymptomatic plants) produce β-lactam and carbapenem antibiotics that play a role in bacterial competition and might give them better fitness in the ecological niche [53]. 

Furthermore, some of the tested *Pectobacterium* strains showed resistance to antibiotics. Under natural conditions, this feature is acquired as a means of protection against bacteriocins secreted by other bacteria with which the genus *Pectobacterium* competes for ecological niche (e.g., the genus *Pseudomonas*). Plants also produce bactericidal compounds, e.g., isothiocyanates. Genetic determinants of the enzymes responsible for their degradation have been found in the chromosomes of many *Pectobacterium* species [20]. Conjugation plasmids are a possible way of acquiring resistance to antibiotics or other bactericidal compounds. In addition, plasmids carry, for example, genes coding heavy metal removal pump systems that allow the survival in the presence of many plant protection compounds. Such plasmids carrying enzymes enabling antibiotic degradation have been described in *P. versatile* SCC1 and *P. zantedeschiae* 9M^T^ strains [5,54]. The ability to take in plasmids and acquire new traits provides an advantage for bacteria in adapting to new environmental conditions. We also decided to check if *Pectobacterium* isolates are susceptible to specific antibiotics. This may pose a serious economic problem in the future. Strains with the Extended Spectrum Betalactamases positive (ESBL+) phenotype are resistant to many β-lactam antibiotics and other chemical compounds, which makes it easier for them to undergo positive selection and persist in flora, and finally spread in the environment. The ESBL coding genes are located on plasmids, which usually also contain genes conditioning resistance to e.g., aminoglycosides, co-trimoxazole, tetracyclines or chloramphenicol [55]. This is currently the most important topic in the field of antimicrobial drug resistance. Build-up of multiresistance carbapenemase-producing microbes provokes questions about the future of treatment of clinical and environmental infections [56,57]. Some of the isolates tested showed resistance or low sensitivity to ampicillin. 

In our recent studies, we have shown that bacteria from the genus *Pectobacterium* produce extracellular membrane vesicles (MVs) harbouring various enzymes, among them β-lactamases. Furthermore, we have shown that MVs produced by ampicillin resistant *P. versatile* strain DPMP190 enable the growth of sensitive *E. coli* strain in the presence of 300 µg mL^−1^ of ampicillin in the medium [58]. The mechanism of secretion of enzymes degrading antibiotics outside the cell via MVs has the key importance of the effective colonisation of an ecological niche and competence with other microorganisms present in it. Thus, the presence on the same plant samples strains of *P. versatile* that are resistant to antibiotics and produce MVs with active β-lactamases and carbapenemases and might allow for the growth of strains that are susceptible to bacteriocins produced by strains co-existing in the same niche. Furthermore, β-lactamases which can cut the lactam ring are able to degrade the homoserine lactones. Therefore, they can play a significant role in disrupting the cell signalling of other bacteria competing for the same niche, for example, hindering their biofilm formation or reducing virulence. In our observations, *P. versatile* occurred on the same plant sample together with *P. carotovorum*, *P. brasiliense*, *P. atrosepticum* or with *P. parvum*. This co-existence could be due to secretion via MVs of enzymes degrading antimicrobial compounds produced by other microorganisms present on the same plant.

Recently, numerous *P. versatile* strains and limited number of *P. brasiliense* and *P. polaris* strains harbouring genetic determinates of beta-lactamases have been described [59]. The large variety of environments from which *P. versatile* strains carrying β-lactamases have been isolated indicates the extraordinary ability of these bacteria to colonise various environments. 

Based on the results of pathogenicity tests, we have demonstrated that strains isolated from asymptomatic plants could macerate plant tissues in laboratory conditions. Furthermore, strains isolated from symptomless plants have a similar ability to macerate plant tissues as strains retrieved from plants with disease symptoms. Except for four strains, all of them were able to macerate plant species other than those from which they have been isolated. Similar observations are widely described in the literature in the case of strains isolated from symptomatic plants or water [3]. 

However, for the strains from symptomatic plants a slightly smaller rot area was observed than for asymptomatic strains (Figure 5). The observed difference between mean rotting area of strains isolated from symptomatic and asymptomatic plants is statistically significant; however, the ranges exhibited by both groups of strains are very similar and overlapping. This difference may be due to the fact that the reference strains and Polish strains derived from plants with disease symptoms were isolated much earlier and have been stored in laboratories at least for 25 years; therefore, it cannot be excluded that they have partially lost their virulence. However, to definitively ascertain whether the strains from symptomatic and asymptomatic plants exhibit a statistically significant difference in virulence, additional tests should be carried out on a larger group of strains. Primarily because in the range of each of the tested species, the strains show a very diverse ability to macerate plant tissues.

Undoubtedly, *Pectobacterium* strains are sensitive to the lack of water in the environment in which they occur. However, they possess the ability to survive in conditions of limited water availability. Some of the strains tested after 48 h of incubation in the condition of limited water availability by adding PEG to the medium were able to resume their growth. This may explain why *Pectobacterium* is able to survive on the vegetables or ornamental plants in warm climates or during transport and storage. We also checked the survivability of tested bacteria in different pH. The most optimal range of pH for growth of *Pectobacterium* spp. was between 5 and 6, which is comparable to pH usually occurring in plant tissues [60,61]. They were also able to cope well at pH 7 and 8. After 48 h of incubation, the strains were also able to adjust to pH 10 and 11 and resumed their growth. Most of the strains could not survive in the environment with pH = 4. Bacteria of the genus *Pectobacterium* grew well in the medium with salinity in the range from 0 to 4%. Above that value, bacteria grew poorly. However, the spot tests indicated that the bacteria remained viable in such extreme conditions. It can be concluded that they were able to arrest their metabolism and survive while awaiting favourable growth conditions. The most resistant to the changes in the pH and salinity conditions was the Moroccan strain *P. brasiliense* DPMP55 isolated from potato. It is worth underlining that ware potatoes from Mediterranean regions are sold frequently and are readily available in European stores during the winter period. Other stress-resistant strains were two isolates of *P. carotovorum* DPMP199 and DPMP200 from Egyptian potatoes and two *P. versatile* isolates from cactus DPMP198 DPMP202. These strains are extremely resistant to the reduced water content in the environment, and thus they have a much greater possibility of surviving long-term transport and storage.

Undoubtedly, the results of adaptation tests and antibiotic resistance indicate that, among the detected *Pectobacterium* species, strains classified as *P. versatile* are the most resistant to antibiotics and are characterised by the ability to secrete β-lactamases and carbapenemases. In addition, of all the strains tested, two strains of *P. versatile* DPMP198 and DPMP202 from the cactus can grow in the most extensive pH range and the lowest water availability, while the *P. versatile* DPMP402 strain is viable in a medium containing from 0–11% NaCl. Thus, the species *P. versatile*, characterised by high genetic and phenotypic variability and high adaptation abilities, has the greatest potential to spread and effectively colonise new environments. It is confirmed by the number of strains isolated from various environments, from plants to water, soil, or insects. This taxon is the most numerous among the strains collected in the collections and among species detected on asymptomatic plants, which was shown in this study.

## 4. Materials and Methods

### 4.1. Bacterial Strains and Growth Conditions

*Pectobacterium* strains used in this study are listed in Table 1. The one hundred and sixty-one bacteria strains were isolated from 149 different symptomless plant samples collected from 1999 up to 2018. They were 108 samples of ware vegetables (Broccoli, Cabbage, Cactus, Carrot, Celery, Fennel, Garlic, Ginger, Leek, Onion, Parsley, Peppers, Potato, Prickly Pear, Rhubarb, Rutabaga, Sugar Beet, Sweet Potato, Tomato, and Zucchini). Of these, 65 were potatoes. In addition, we have tested 41 samples of ornamental and herbaceous plant species (Alpine violet, Bean, Beetroot, Bittersweet, Black nightshade, Cactus, Dieffenbachia, Iris, Kalonchoe, Opuntia, Rose, Pigweed). 

Plants were originated from 25 countries (Armenia, Belgium, Brasil, Cyprus, Egypt, Finland, France, Germany, Georgia, Israel, Italy, Japan, Kazakhstan, Morocco, Poland, Portugal, the Netherlands, New Zealand, Norway, Serbia, Spain, Tenerife, Tunisia, UK, USA).

Additionally, 26 strains isolated from symptomatic plants were used as a reference for comparison purposes.

To isolate new bacterial strains, about 15 cm^2^ of plant area were suspended in 15 mL of 0.96% NaCl and were homogenised by grinding in a mortar and pestle. After 2 h of preincubation with shaking at 28 °C, the samples were serially diluted. Next, 100 μL aliquots of serial dilutions of homogenate (10^−2^, 10^−3^, 10^−4^ and 10^−5^) were plated on a Crystal Violet Pectate (CVP) medium [62] and incubated for 48 h at 28 °C. Colonies that formed cavities were restreaked on CVP medium and incubated as previously. For long time storage, all isolates were kept as frozen stocks at −80 °C To prepare frozen stocks, single bacterial colonies were transferred to 7 mL of TSB medium and then grown for 48 h at 28 °C with shaking. Next, 500 µL of bacterial culture was mixed with 500 µL sterile 80% glycerol.

### 4.2. Phenotypic Characteristics of Newly Isolated Strains

#### 4.2.1. Plant Tissue Maceration Assays

The ability to macerate plant tissue was determined for 26 pectinolytic strains newly isolated from asymptomatic plants and 21 strains originating from plants with disease symptoms. For the pathogenicity assays, bacteria of various origins were chosen (Table S1).

Leaves of Irises and Chicory were washed with distilled water. An overnight bacterial culture in the TSB medium was diluted with physiological saline solution to an optical density of 0.5 McF. A one-centimeter cut was made on the surface of the leaf across the conductive bundle, and 25 μL of the bacterial suspension was then dripped into the cut. Inoculated leaves (in three replicates per strain) were placed in plastic sampling bags with sealing strips lined with paper towels soaked in sterile distilled water. Such prepared samples were incubated at 28 °C for 48 h. After 24 and 48 h of inoculation, the average area of rotten tissue was calculated. As a negative control, leaves were inoculated with 25 μL of sterile water.

Potato tubers were thoroughly washed, and their surface was disinfected in a 1% hypochlorite solution bath. The overnight bacterial culture in TSB medium was diluted with Ringer solution to an optical density of 0.5 McF. The tubers were punctured with pipette tips containing 50 μL of the suspension. The tips were left in the tubers. A total of three punctures were made on each tuber. Each strain was tested on 3 tubers in triplicates. Inoculated tubers were placed in plastic boxes. In addition, 500 mL of distilled water was poured into the bottom of the boxes to achieve relative humidity above 90%. After 72 h of incubation at 28 °C, the diameter of the macerated tissue was measured. As a negative control, pipette tips inserted into tubers contained sterile water rather than a bacterial suspension.

#### 4.2.2. Adaptation to Various Environmental Conditions

The ability to grow in various environmental conditions such as different pH, salinity and water availability was determined for 22 pectinolytic strains newly isolated from asymptomatic plants and 13 strains originating from plants with disease symptoms. (Table 3, Table 4 and Table 5). 

The pH effect on bacterial growth was studied in TSB medium under pH values of 4, 5, 6, 7, 8, 9, 10 and 11, respectively. 

The ability to grow in various salinity conditions was conducted in TSB medium supplemented with 0 g L^−1^, 10 g L^−1^, 20 g L^−1^, 30 g L^−1^, 40 g L^−1^, 50 g L^−1^, 60 g L^−1^, 70 g L^−1^ NaCl and 80 g L^−1^.

The tolerance for limited water availability was estimated in TSB medium supplemented with 0.0 g L^−1,^ 50.0 g L^−1^, 75.0 g L^−1^, 100.0 g L^−1^, 200.0 g L^−1^, 300.0 g L^−1^, 400.0 g L^−1^ and 500.0 g L^−1^ of polyethylene glycol (PEG).

The assays were performed in 96-well titration plates. The 200 μL of TSB medium with various pH, salinity and PEG concentration was inoculated with 5 μL of bacterial suspension with an optical density of 0.5 McF. The plates were incubated with shaking at 28 °C. The absorbance readings at 600 nm were made after 0, 6, 24 and 48 h of incubation, using the Infinite M200 Pro (Tecan). The experiments with two replicates were performed twice.

To assess the viability of bacteria under different pH conditions, salinity and water availability after 48 h of incubation, spot tests were performed on TSA medium. Furthermore, 5 μL of bacterial culture was withdrawn from each well and dropped onto a plate, which was then incubated for 48 h at 28 °C. Additionally, after performing the spot test, 20 μL of 0.02% resazurin solution was added to each well, and the plate was incubated for 24 h at 28 °C. The result was read colorimetrically. During bacterial metabolism, purple resazurin is transformed to pink resorufin (pH = 6.5). Under acidic conditions, resorufin takes on a yellow colour (pH = 3.8). This allows for a colorimetric reading of the presence of acid metabolism products in the medium.

#### 4.2.3. Antibiotic Susceptibility Assay

The antibiotic susceptibility of 34 selected *Pectobacterium* strains (31 isolated from asymptomatic plants and 3 from symptomatic) was tested by a standard disc diffusion method. In addition, 100 μL of a bacterial suspension with an optical density of 0.5 McF was spread on a Mueller–Hinton (MH) medium. Antibiotic discs containing ampicillin 10 μg, erythromycin 15 μg, gentamicin 250 μg, kanamycin 30 μg, streptomycin 10 μg, tetracycline 15 μg were then applied (Emapol). The plates were incubated for 24 h at 28 °C. Next, the zone of bacterial growth inhibition around the antibiotic disc was assessed. 

Moreover, for strains that showed resistance to at least one antibiotic in the disc diffusion test, the production of beta-lactamases and carbapenemases was determined with application of two chromogenic media, ESBL (extended-spectrum beta-lactamases) chromagar and KPC (Klebsiella pneumoniae carbapenemase) plates (GRASSO). The control *Escherichia coli* NCTC 13351 and *Klebsiella pneumoniae* BAA-1705, which grow on the above-mentioned media and have ESBL and KPC resistance mechanisms, have been used.

#### 4.2.4. Statistical Analysis

The analysis of differences between the groups was performed using the ANOVA with Welch corrections for nonhomogeneous variances criterion followed by a post-hoc Games–Howell analysis in R [63].

### 4.3. Molecular Identification of Newly Isolated Strains

#### 4.3.1. DNA Isolation

For DNA isolation, bacterial strains were grown overnight in 7 mL of Tryptic Soy Broth (TSB) at 28 °C with shaking. Cells were harvested by centrifugation and resuspended in 500 µl TE buffer (50 mM Tris/HCl, 40 mM EDTA, pH 8.0). Afterwards, the cell lysis and nucleic acids extraction were carried out according to the protocol proposed by a Joint Genome Institute for bacterial DNA isolation using CTAB [64] followed by the RNA digestion using Turbo RNase (Ambion). DNA quantity and quality were assessed first using a NanoDrop Spectrophotometer and later with agarose gel electrophoresis.

#### 4.3.2. PCR Amplification Sequencing and Phylogenetic Analysis

DNA amplification was performed in 25 µL reaction volumes using PCR Master Mix (Thermo Scientific; K0171) according to the manufacturer’s instruction. Amplification was performed using a T100 Bio-Rad thermocycler. The amplified products were separated in 1.5% (*w/v*) agarose gel at 100 V for 40 min in 0.5xTAE buffer and visualised with UV light after staining in ethidium bromide (0.5 g mL^−1^).

Species specific PCR reactions were performed according to the previously described protocols [29,30,31,32,35]. The genetic diversity of *Pectobacterium* and *Dickeya* strains was analysed with a fingerprinting method, Enterobacterial Repetitive Intergenic Consensus Polymerase Chain Reaction (ERIC-PCR), according to the procedure described by Versalovic et al. [34]. For amplification and sequencing of the *recA* gene fragment, primers previously described were used [38]. Sequencing was carried out using an ABI PRISM DNA Sequencer (PerkinElmer) according to the manufacturer’s manual. Both strands were sequenced using the forward and reverse PCR primers. 

Furthermore, the obtained sequences were subjected to BLAST sequence similarity search analysis to identify the nearest taxa. The obtained sequences of the *recA* gene and sequences of the closest representative taxa that belong to *Pectobacterium* and *Dickeya* were aligned using an MAFFT algorithm in Geneious v9.1.8. [65]. The phylogenetic analysis was performed with the MEGA v. X software, (www.megasoftware.net, accessed on 20 May 2022), and trees were constructed using the Maximum Likelihood algorithm and Hasegawa–Kishino–Yano models selected on the model test module implemented in MEGA. Bootstrap analysis with 1000 replications was performed to assess the robustness of the clusters. 

## 5. Conclusions

Our research proved that bacteria of the genus *Pectobacterium* isolated from asymptomatic ware vegetables and ornamental plants can cause maceration of plant tissues of more than one plant species. These strains were also able to adapt to extreme environmental conditions, such as low water accessibility, acidic and alkaline pH, and high salinity. The antibiograms’ results showed that some of these bacteria had resistance to betalactams, macrolides, aminoglicosides and tetracyclines; in addition, they might possess beta-lactamases of extended spectrum or carbapenemases. 

Undoubtedly, we have demonstrated that *Pectobacterium* strains isolated from symptomless vegetables and ornamental plants traded internationally show a high potential for adaptation to adverse environmental conditions and ability to change the host plant. As a result, they may contribute to the success of the genus *Pectobacterium* and accelerate its spreading between different climatic zones and facilitate the colonisation of different ecological niches.

The most important claim we want to make, which has not been previously described, is that internationally traded ware vegetables, ornamental plants and herbs which do not undergo strict phytosanitary control serve as a significant transmission medium responsible for global distribution of widely observed species such as *P. versatile*, which remains the most frequently isolated pathogenic taxon from *Pectobacterium* genus. Thus, we would like to bring the attention of the scientific community to this underexplored area, and look beyond pathogen presence in seed material, towards mass market wares and organic waste which become more and more important in the age of sustainable agriculture and a zero waste lifestyle.

## Figures and Tables

**Table 1:** Host plant, geographical origin, year of isolation, *recA* PCR, sequence accession numbers specific PCR applied, and ERIC profile of the studied *Pectobacterium* isolates.

Plant Sample No.	Strain Designation	Isolation Source	Geographic Origin of Sample	Year of Isolation	Darrasse PCR/ Nassar PCR (P/D)	*Reca* PCR Sequence Accession No	^#^Species Specific PCR	ERIC Profile
*Pectobacterium atrosepticum*								
mw1	IFB5094	Garlic	Poland	2002	P+	+ This work	Pba	A1
mw2	IFB5095	Parsley	Poland	2002	P+	+ This work	Pba	A1
39	^a^ DPMP134	Potato tuber	Belgium	2015	P+	+	Pba	A1
45	DPMP148	Parsley	Poland	2015	P+	+ This work	Pba	A2
73	DPMP275	Potato tuber	Norway	2016	P+	+ This work	Pba	A1
73	^j^ DPMP278	Potato tuber	Norway	2016	P+	+	Pba	A1
61	DPMP226	Ginger	Poland	2016	P+	+	Pba	A3
61	DPMP227	Ginger	Poland	2016	P+	+	Pba	A3
92	DPMP330	Weed	Poland	2016	P+	+	Pba	A1
92	DPMP332	Weed	Poland	2016	P+	+	Pba	A1
96	DPMP340a	Fennel	Poland	2016	P+	+	Pba	A1
98	^b^ DPMP350	Potato stem	Poland	2016	P+	+	Pba	A1
98	DPMP366	Potato stem	Poland	2016	P+	+	Pba	A1
101	DPMP371	Potato tuber	United Kingdom	2016	P+	+	Pba	A3
106	DPMP381	Sugar beet	Poland	2016	P+	+	Pba	A1
125A	DPMP442	Potato stem	Poland	2017	P+	+	Pba	A1
149	DPMP623	Diffenbachia	Poland	2018	P+	+	Pba	A1
147	DPMP617	Diffenbachia	Poland	2018	P+	+	Pba	A1
155	^c^ DPMP634	Potato tuber	Kazakhstan	2018	P+	+	Pba	A2
*75B1	*DPMP759	**Potato tuber**	**Poland**	**1996**	**P+**	**+**	**Pba**	**A3**
*16A1	*IFB5050	**Potato stem**	**Poland**	**1996**	**P+**	**AY217078**	**Pba**	**A1**
	*SCRI1043	**Potato stem**	**Scotland UK**	**1985**	**P+**	**BX950851**	**Pba**	**A3**
	*CFBP1526	**Potato**	**United Kingdom**	**1957**	**P+**	**JQHK00000000**	**Pba**	**A1**
*57A1	*IFB5205	**Potato stem**	**Poland**	**1996**	**P+**	**+ This work**	**Pba**	**A1**
*Pectobacterium brasiliense*								
2	DPMP17	Potato tuber	Cyprus	2013	P+	+ This work	-	B3
6	DPMP32	Cabbage	Poland	2015	P+	+ This work	Pbr	B1
7	DPMP33	Leek	Poland	2015	P+	+	Pbr	B1
16	^d^ DPMP55	Potato tuber	Morocco	2013	P+	+	Pbr	B1
21	DPMP81	Potato tuber	Spain Tenerife	2014	P+	+	Pbr	B2
38	DPMP132	Sweet Potato	USA	2015	P+	+	Pbr	B2
25	DPMP135	Rhubarb	Poland	2015	P+	+	Pbr	B2
44	^e^ DPMP152	Potato tuber	Morocco	2015	P+	+	Pbr	B2
46	DPMP153	Potato tuber	Israel	2015	P+	+ This work	Pbr	B2
46	DPMP154	Potato tuber	Israel	2015	P+	+	Pbr	B2
60	DPMP224	Potato tuber	Portugal	2016	P+	+ This work	Pbr	B2
66	DPMP255	Celery	Poland	2016	P+	+	Pbr	B2
101	DPMP372	Potato tuber	United Kingdom	2016	P+	+	Pbr	B2
103	DPMP374	Potato tuber	Brasil	2016	P+	+ This work	Pbr	B2
103	DPMP375	Potato tuber	Brasil	2016	P+	+	Pbr	B2
103	DPMP377	Potato tuber	Brasil	2016	P+	+	Pbr	B2
103.2	DPMP378	Potato tuber	Brasil	2016	P+	+	Pbr	B2
111	^f^ DPMP394	Sugar beet	Poland	2016	P+	+ This work	-	B2
112	^g^ DPMP396	Bittersweet	Poland	2016	P+	+ This work	-	B2
156	DPMP678	Rhubarb	Poland	2018	P+	+	Pbr	B4
156	DPMP679	Rhubarb	Poland	2018	P+	+	Pbr	B4
156	DPMP680	Rhubarb	Poland	2018	P+	+ This work	Pbr	B4
156	DPMP681	Rhubarb	Poland	2018	P+	+	Pbr	B4
157	DPMP682	Potato tuber	The Netherlands	2018	P+	+	Pbr	B4
29	DPMP120	Zucchini	Poland	2016	P+	+ This work	Pbr	B2
mkw16	MKW16	Potato tuber	Cyprus	2013	P+	+ This work	Pbr	B3
mkw33	MKW33	Potato tuber	Morocco	2013	P+	+ This work	Pbr	B1
*mw3	**IFB5258**	**Sugar beet**	**Poland**	**2002**	**P+**	**KP762589**	**Pbr**	**B2**
*MN9	*IFB5164	**Parsley**	**Poland**	**2002**	**P+**	**+ This work**	**Pbr**	**B3**
*110-6B	*IFB5369	**Potato**	**Poland**	**2011**	**P+**	**KP762587**	**Pbr**	**B2**
	*LMG2137^T^	**Potato**	**Brazil**	**1999**	**P+**	**JQOE01000000**	**Pbr**	**B4**
*Pectobacterium carotovorum*								
10	DPMP60	Prickly pear	Tunisia	2016	P+	+	-	C3
44	^e^ DPMP146	Potato tuber	Morocco	2015	P+	+	-	C1
51.1	DPMP189	Potato tuber	Egypt	2015	P+	+ This work	-	C2
51.5	DPMP199	Potato tuber	Egypt	2016	P+	+ This work	-	C2
51.3	DPMP200	Potato tuber	Egypt	2016	P+	+ This work	-	C2
51.4	DPMP262	Potato tuber	Egypt	2016	P+	+	-	C1
77	DPMP292	Onion	Poland	2016	P+	+	-	C1
87	DPMP323	Prickly pear	Italy	2016	P+	+	-	C3
98	DPMP346	Potato stem	Poland	2016	P+	+	-	C2
98	DPMP351	Potato stem	Poland	2016	P+	+	-	C2
99.1	DPMP356	Potato tuber	Poland	2016	P+	+	-	C2
99.3	DPMP357	Potato tuber	Poland	2016	P+	+	-	C2
99.3	DPMP358	Potato tuber	Poland	2016	P+	+	-	C2
100	^h^ DPMP369	Potato tuber	Cyprus	2016	P+	+	-	C3
102	DPMP373	Broccoli	Poland	2016	P+	+	-	C2
109	DPMP389	Potato tuber	Japan	2016	P+	+	-	C1
113	DPMP398	Sugar beet	Poland	2016	P+	+ This work	-	C3
113	DPMP399	Sugar beet	Poland	2016	P+	+ This work	-	C3
113	DPMP400	Sugar beet	Poland	2016	P+	+ This work	-	C3
113	DPMP401	Sugar beet	Poland	2016	P+	+ This work	-	C3
119	DPMP417	Leek	Poland	2016	P+	+	-	C1
119	DPMP418	Leek	Poland	2016	P+	+	-	C1
109	DPMP421	Potato tuber	Japan	2016	P+	+	-	C1
141	DPMP598	Potato tuber	Georgia	2017	P+	+	-	C1
142	DPMP607	Alpine violet	Poland	2018	P+	+	-	C2
143	DPMP608	Alpine violet	Poland	2018	P+	+	-	C2
145	DPMP613	Alpine violet	Poland	2018	P+	+	-	C1
146	DPMP615	Diffenbachia	Poland	2018	P+	+	-	C1
147	DPMP616	Diffenbachia	Poland	2018	P+	+	-	C1
147	DPMP618	Diffenbachia	Poland	2018	P+	+	-	C2
148	DPMP619	Diffenbachia	Poland	2018	P+	+	-	C2
148	DPMP622	Diffenbachia	Poland	2018	P+	+	-	C1
155	^c^ DPMP631	Potato tuber	Kazakhstan	2018	P+	+	-	C1
*134A2	*DPM510	**Potato stem**	**Poland**	**1996**	**P+**	**AY264792**	**-**	**C4**
	*LMG2401	**Carot**	**USA**	**1967**	**P+**	**+**	**-**	**C1**
	*LMG2404^T^	**Potato**	**Danemark**	**1952**	**P+**	**JQHJ00000000**	**-**	**nt**
*Pectobacterium odoriferum*								
4	DPMP27	Celery	Poland	2015	P+	+ This work	-	O1
78	DPMP293	Celery	Poland	2016	P+	+ This work	-	O1
**MN6**	*IFB5295	**Carrot**	**Poland**	**1999**	**P+**	**AY264791**	**-**	**O1**
**L9**	*IFB5300	**Leek**	**Poland**	**1995**	**P+**	**KF704816**	**-**	**O1**
	*CFBP1878^T^	**Chicory**	**France**	**1979**	**P+**	**KF704811**	**-**	**O1**
*Pectobacterium parmentieri*								
40	DPMP136	Potato tuber	Poland	2015	P+	+ This work	Ppar	Pa1
96	DPMP340b	Fennel	Poland	2016	P+	+	Ppar	Pa1
60	DPMP225	Potato tuber	Portugal	2016	P+	+	Ppar	Pa1
98.2	DPMP347	Potato stem	Poland	2016	P+	+	Ppar	Pa1
99.1	^k^ DPMP353	Potato tuber	Poland	2016	P+	+ This work	Ppar	Pa2
99.1	DPMP354	Potato tuber	Poland	2016	P+	+	Ppar	Pa2
99.3	DPMP355	Potato tuber	Poland	2016	P+	+	Ppar	Pa2
99.1	DPMP362	Potato tuber	Poland	2016	P+	+	Ppar	Pa2
99.1	DPMP363	Potato tuber	Poland	2016	P+	+	Ppar	Pa1
99.2	DPMP364	Potato tuber	Poland	2016	P+	+	Ppar	Pa1
100	^h^ DPMP370	Potato tuber	Cyprus	2016	P+	+	Ppar	Pa1
109	DPMP390	Potato tuber	Japan	2016	P+	+	Ppar	Pa1
	*SCC3193	**Potato stem**	**Finland**	**1980s**	**P+**	**CP003415**	**Ppar**	**Pa1**
	*IFB5322	**Potato sten**	**Poland**	**1996**	**P+**	**AY217080**	**Ppar**	**Pa1**
*Pectobacterium parvum*								
mkw18	^i^ DPMP20	Potato tuber	Cyprus	2013	P+	+ This work	-	Pv1
20	DPMP78	Peppers	Spain Tenerife	2014	P+	+	-	Pv1
53	DPMP223	Ginger	Poland	2016	P+	+	-	Pv1
	*so421	**Potato stem**	**Finland**	**2005**	**P+**	**OANP00000000**	**-**	**Pv1**
**38A1**	*IFB5220	**Potato stem**	**Poland**	**1996**	**P+**	**PHSZ00000000**	**-**	**Pv1**
*Pectobacterium polaris*								
73	^j^ DPMP280	Potato tuber	Norway	2016	P+	+	-	Po1
76	DPMP286	Potato tuber	Finland	2016	P+	+ This work	-	Po1
76	DPMP290	Potato tuber	Finland	2016	P+	+	-	Po1
106	DPMP380	Sugar beet	Poland	2016	P+	+ This work	-	Po2
112	^g^ DPMP397	Bittersweet	Poland	2016	P+	+ This work	-	Po2
111	^f^ DPMP403	Sugar beet	Poland	2016	P+	+ This work	-	Po3
114	DPMP404	Rutabaga	Poland	2016	P+	+ This work	-	Po3
114	DPMP405	Rutabaga	Poland	2016	P+	+ This work	-	Po3
16	^d^ DPMP730	Potato tuber	Morocco	2013	P+	+	-	Po4
mw10	IFB5223	Black nightshade	Poland	2002	P+	+ This work	-	Po3
mkw36	MKW36	Potato tuber	Morroco	2013	P+	+ This work	-	Po4
**104B2**	*IFB5252	**Potato tuber**	**Poland**	**1996**	**P+**	**KU510113**	**-**	**Po3**
**119A1**	*IFB5222	**Potato stem**	**Poland**	**1996**	**P+**	**KU510110**	**-**	**Po4**
**129A1**	*IFB5225	**Potato stem**	**Poland**	**1996**	**P+**	**KU510111**	**-**	**Po4**
	*NBIO1006^T^	**Potato tuber**	**Norway**	**2010**	**P+**	**CP017481**	**-**	**Po3**
*Pectobacterium versatile*								
k19	IFB5176	Cabbage	Poland	1999	P+	+ This work	-	V2
p36	IFB5178	Parsley	Poland	1999	P+	+ This work	-	V2
p42	IFB5179	Parsley	Poland	1999	P+	+ This work	-	V2
ce42	IFB5181	Celery	Poland	1999	P+	+ This work	-	V2
mw6	IFB5212	Rose	Poland	2002	P+	+ This work	-	V2
mw57	IFB5215	Garlic	Germany	1999	P+	+ This work	-	V1
mw8l	IFB5213	Bittersweet	Poland	2002	P+	+ This work	-	V1
mw8b	IFB5214	Bittersweet	Poland	2002	P+	+ This work	-	V1
mkw18	^i^ MKW18	Potato tuber	Cyprus	2013	P+	+ This work	-	V2
mkw32	MKW32	Potato tuber	Morocco	2013	P+	+ This work	-	V1
5	DPMP28	Leek	Poland	2015	P+	+	-	V2
9	DPMP35	Leek	Poland	2015	P+	+	-	V2
27.5p	DPMP98	Potato tuber	Israel	2015	P+	+ This work	-	V4
27.5p	DPMP100	Potato tuber	Israel	2015	P+	+ This work	-	V4
27.3p	DPMP102	Potato tuber	Israel	2015	P+	+	-	V3
32	DPMP114	Potato tuber	Morocco	2013	P+	+	-	V3
29	DPMP106	Zucchini	Poland	2016	P+	+	-	V3
28	DPMP105	Iris	Poland	2016	P+	+ This work	-	V3
28	DPMP108	Iris	Poland	2016	P+	+ This work	-	V3
31	DPMP112	Iris	Poland	2016	P+	+ This work	-	V3
39	^a^ DPMP133	Potato tuber	Belgium	2015	P+	+	-	V1
42	DPMP140	Potato tuber	Poland	2015	P+	+	-	V1
44	^e^ DPMP155	Potato tuber	Morocco	2015	P+	+	-	V3
45	DPMP156	Parsley	Poland	2015	P+	+ This work	-	V3
29	DPMP181	Zucchini	Poland	2016	P+	+ This work	-	V2
52	DPMP198	Cactus	Poland	2016	P+	+ This work	-	V2
52	DPMP202	Cactus	Poland	2016	P+	+ This work	-	V2
54	DPMP204	Potato tuber	France	2016	P+	+ This work	-	V3
57	DPMP217	Potato tuber	Spain	2016	P+	+	-	V3
58	DPMP228	Potato tuber	Spain	2016	P+	+ This work	-	V3
62	DPMP234	Onion	Poland	2016	P+	+ This work	-	V3
65.1p	DPMP238	Potato tuber	Cyprus	2016	P+	+ This work	-	V2
65.2.p	DPMP240	Potato tuber	Cyprus	2016	P+	+	-	V2
65.1p	DPMP248	Potato tuber	Cyprus	2016	P+	+ This work	-	V2
67	DPMP256	Potato stem	Poland	2016	P+	+ This work	-	V3
80t	DPMP299	Tomato	Poland	2016	P+	+ This work	-	V2
81p	DPMP300	Potato tuber	Poland	2016	P+	+ This work	-	V2
96	DPMP337	Fenel	Poland	2016	P+	+	-	V1
98	^b^ DPMP344	Potato stem	Poland	2016	P+	+ This work	-	V2
99.1	^k^ DPMP352	Potato tuber	Poland	2016	P+	+ This work	-	V1
94	DPMP334	Bean	Poland	2016	P+	+	-	V4
95	DPMP335	Peppers	Morocco	2016	P+	+ This work	-	V4
106	DPMP383	Sugar beet	Poland	2016	P+	+	-	V2
108	DPMP387	Beetroot	Poland	2016	P+	+ This work	-	V2
114	DPMP402	Beetroot	Poland	2016	P+	+ This work	-	V2
130	DPMP500	Bittersweet	Poland	2002	P+	+	-	V1
130	DPMP501	Bittersweet	Poland	2002	P+	+ This work	-	V1
137	DPMP546	Iris	Poland	2016	P+	+ This work	-	V3
155	^c^ DPMP632	Potato tuber	Kazakhstan	2018	P+	+ This work	-	V4
155	^c^ DPMP633	Potato tuber	Kazakhstan	2018	P+	+ This work	-	V4
**61A1**	*IFB5206	**Potato stem**	**Poland**	**1996**	**P+**	**MK024782**	**-**	**V1**
**75B5**	*IFB5208	**Potato tuber**	**Poland**	**1996**	**P+**	**MK024779**	**-**	**V1**
**25A3**	*IFB5169	**Potato stem**	**Poland**	**1996**	**P+**	**MK024780**	**-**	**V4**
**143A1**	*IFB5266	**Potato stem**	**Poland**	**1996**	**P+**	**MK024781**	**-**	**V1**
	*IFB5462	**Potato tuber**	**Poland**	**1996**	**P+**	**KU510133**	**-**	**V1**
	*SCC1	**Potato tuber**	**Finland**	**1982**	**P+**	**CP021894**	**-**	**V2**
*Pectobacterium zantedeschiae*								
	*9M^T^	**Calla lily**	**Poland**	**2018**	**P+**	**MH367240**	**-**	
*Dickeya dadantii*								
150	DPMP624	Diffenbachia	Poland	2018	D+	+	-	D1
151	DPMP625	Diffenbachia	Poland	2018	D+	+ This work	-	D1
152	DPMP626	Diffenbachia	Poland	2018	D+	+	-	D1
153	DPMP627	Diffenbachia	Poland	2018	D+	+	-	D1
	*3937	**African violet**	**France**	**1980**	**D+**	**CP002038**	**-**	**D1**

* Strains isolated from plants with disease symptoms used as reference in this research; #Pba positive result of PCR with Eca1/Eca2 primers [29]; Pbr positive result of PCR with Br1f/L1r primers [30]; Ppar positive result of PCR with PhF/PhR primers [31]; ^a^—potato tuber sample Liege 2/2015; ^b^—potato stem Lodyga3/16; ^c^—potato tuber sample Szymkent 215, ^d^—potato tuber sample Maroko13, ^e^—potato tuber sample Maroko15; ^f^—Sugar beet sample BC16; ^g^—Bittersweet sample SD16; ^h^—potato tuber sample Cypr103; ^i^—potato tuber sample Cypr4/1; Darrasse—a PCR test for *Pectobacterium* spp. using Y1, Y2 primers [32]; ^j^—potato tuber sample Norwegia28; ^k^—potato tuber sample Patków99; ^T^—type strain; P+—the strain was identified as *Pectobacterium*; Nassar PCR with primers specific for *Dickeya* genus [35] D+—the strain was identified as *Dickeya.*

**Table 2:** List of *Pectobacterium* strains classified into different species that were co-isolated from the same plant sample.

Plant Sample No/Name	Host Plant	Geographic Origin of Plant	Detected *Pectobacterium* species	Year of Isolation
111/BC16	Sugar beet	Poland	*P. brasiliense* DPMP394 *P. polaris* DPMP403	2016
112/SD16	Bittersweet	Poland	*P. brasiliense* DPMP396 *P. polaris* DPMP397	2016
155/Maroko2013	Potato tuber	Morocco	*P. brasiliense* DPMP55 *P. polaris* DPMP730	2013
39/Łodyga3/16	Potato stem	Poland	*P. atrosepticum* DPMP350 *P. versatile* DPMP344	2016
39/Liege 2/2015	Potato tuber	Belgium	*P. atrosepticum* DPMP134 *P. versatile* DPMP133	2015
73/Norwegia28	Potato tuber	Norway.	*P. atrisepticum* DPMP278 *P. polaris* DPMP280	2016
99.1/Patków 99	Potato tuber	Poland	*P. versatile* DPMP352 *P. parmentieri* DPMP353	2016
100/Cypr103	Potato tuber	Cyprus	*P. parmentieri* DPMP370 *P. carotovorum* DPMP369	2016
mkw18/Cypr 4/1	Potato tuber	Cyprus	*P. parvum* DPMP20 *P. versatile* MKW18	2013
98/Szymkent215	Potato tuber	Kazakhstan	*P. atrosepticum* DPMP634 *P. versatile* DPMP632 and DPMP633 *P. carotovorum* DPMP631	2013
44/Maroko2015	Potato tuber	Morocco	*P. brasilense* DPMP152 *P. carotovorum* DPMP146 *P. versatile* DPMP155	2015

**Table 3:** Measurement of pH effect on bacteria growth.

pH Value								
Strain	4	5	6	7	8	9	10	11
*P. atrosepticum*								
DPMP275	X	OPT						
DPMP442	X	OPT						
*IFB5050			OPT					
*SCRI1043	X		OPT					X
*ICMP1526^T^					OPT			
*P. brasiliense*								
DPMP55			OPT					
DPMP224	X	OPT						
DPMP372	X		OPT					
DPMP374	X		OPT					
DPMP396	X			OPT				X
DPMP680	X	OPT						
*IFB5369	X		OPT					X
*LMG21371^T^	X	X			OPT			
*P. carotovorum*								
DPMP199	V		OPT					
DPMP200	V		OPT					
DPMP323	X	OPT						
DPMP399	X	OPT						
*DPMP510	X	OPT						
*P. parmentieri*								
DPMP390	X		OPT					
* IFB5322	X		OPT					
* SCC3193	X		OPT					
*P. parvum*								
DPMP20	X			OPT				
*IFB5220	X		OPT					
*P. polaris*								
DPMP286		OPT						
DPMP403	X		OPT					X
DPMP397	X	OPT						
*IFB5222	X				OPT			
*IFB5252		OPT						
*P. versatile*								
DPMP198		OPT						
DPMP202		OPT						
*IFB5266	X	OPT						
*IFB5258	X		OPT					
DPMP633	X	OPT						
*DPMP5169	X		OPT					
*P. zantedeschiae*								
*9M^T^	X	OPT						

The symbol “X” means no bacterial growth in the spot test, “V” means the presence of viable bacterial cells in spot test. The “OPT” symbol indicates the optimum at which the strain reached the highest OD value. * reference strains and strains that were isolated from plants with disease symptoms. ^T^—Type Strain. The following colours indicate average OD ranges of two measurements. Black: <0.03, grey: 0.03–0.3, blue: 0.3–0.7, white: >0.7.

**Table 4:** Growth in various salinity conditions.

Concentration of Sodium Chloride [%]									
Strain	0%	1%	2%	3%	4%	6%	7%	8%	11%
*P. atrosepticum*									
DPMP275		OPT					V	V	V
DPMP442		OPT				V	V	X	X
*IFB5050		OPT						X	X
*SCRI1043	OPT								
*ICMP1526^T^	OPT					X	X	X	X
*P. brasiliense*									
DPMP55		OPT							V
DPMP224	OPT							V	V
DPMP372		OPT					V	V	V
DPMP374		OPT					V	V	V
DPMP396					OPT		V	V	V
DPMP680		OPT				V	V	V	X
* **IFB5369**	OPT					V	V	V	X
*LMG21371^T^		OPT				V	V	V	X
*P. carotovorum*									
DPMP199				OPT			V	V	V
DPMP200		OPT					V	V	X
DPMP323		OPT				V	V	V	X
DPMP399	OPT						V	V	V
*DPMP510				OPT			X	X	X
*P. parmentieri*									
DPMP390		OPT			X	X	X	X	X
*IFB5322		OPT			X	X	X	X	X
*SCC3193		OPT			X	X	X	X	X
*P. parvum*									
DPMP20	OPT						V	V	X
*IFB5220		OPT				V	V	V	X
*P. polaris*									
DPMP286	OPT					V	V	V	X
DPMP403	OPT						V	V	X
DPMP397	OPT							V	X
*IFB5222		OPT					V	X	X
*IFB5252		OPT						V	V
*P. versatile*									
DPMP198	OPT							V	X
DPMP202	OPT						V	V	X
IFB402		OPT						V	V
DPMP633	OPT						V	V	X
*IFB5266	OPT					V	V	V	V
*IFB5169				OPT			X	X	X
*P. zantedeschiae*									
*9M^T^	OPT						V	V	X

The symbol “X” means no bacterial growth in the spot test, “V” means the presence of viable bacterial cells in a spot test. The “OPT” symbol indicates the optimum at which the strain reached the highest OD value. * reference strains and strains that were isolated from plants with disease symptoms. ^T^—Type Strain. The average OD ranges are highlighted in the same color as in Table 3.

**Table 5:** The tolerance for limited water availability.

Concentration Polyethylene Glycol [g/L]								
Strain	0 g/L	50 g/L	75 g/L	100 g/L	200 g/L	300 g/L	400 g/L	500 g/L
*P. atrosepticum*								
DPMP275	OPT				V	X	X	X
DPMP442	OPT				X	X	X	X
*IFB5050						X	X	X
*SCRI1043		OPT				X	X	X
*CFBP1526^T^						X	X	X
*P. brasiliense*								
DPMP55	OPT					V	X	X
DPMP224	OPT				V	X	X	X
DPMP372	OPT				V	X	X	X
DPMP374	OPT				V	X	X	X
DPMP396	OPT				V	X	X	X
DPMP680	OPT				V	X	X	X
* **IFB5369**	OPT				V	X	X	X
*LMG21371^T^	OPT					X	X	X
*P. carotovorum*								
DPMP199	OPT						V	X
DPMP200	OPT						V	X
DPMP323	OPT				X	X	X	X
DPMP399	OPT				V	X	X	X
*DPMP510	OPT				X	X	X	X
*P. parmentieri*								
DPMP390	OPT				X	X	X	X
*IFB5322	OPT				X	X	X	X
*SCC3193	OPT				X	X	X	X
*P. parvum*								
DPMP20	OPT				X	X	X	X
*IFB5220	OPT				X	X	X	X
*P. polaris*								
DPMP286	OPT				V	X	X	X
DPMP403	OPT				V	X	X	X
DPMP397	OPT				X	X	X	X
*IFB5222	OPT						X	X
*IFB5252	OPT						V	V
*P. versatile*								
DPMP198	OPT						V	X
DPMP202		OPT						V
DPMP402	OPT				V	V	X	X
DPMP633	OPT				X	X	X	X
*IFB5169	OPT				X	X	X	X
*IFB5266	OPT				V	X	X	X
*P. zantedeschiae*								
*9M^T^	OPT				V	V	X	X

The symbol “X” means no bacterial growth in the spot test, “V” means the presence of viable bacterial cells in spot test. The “OPT” symbol indicates the optimum at which the strain reached the highest OD value. * reference strains and strains that were isolated from plants with disease symptoms. ^T^—Type Strain. The average OD ranges are highlighted in the same colour as in Table 3.

**Table 6:** Antibiotic susceptibility results.

Strain	Antibiotics Susceptibility						ESBL Type Resistance Assay	Carbapenemase Resistance Assay
	Ampicillin 10 µg	Erythromycin 15 µg	Gentamicin 300 µg	Kanamycin 30 µg	Streptomycin 100 µg	Tetracycline 15 µg		
	Zone of growth inhibition [mm]							
*P. atrosepticum*								
DPMP134	0	0	31	35	20	25	-	-
DPMP275	30	16	30	24	16	25	-	-
DPMP366	12	19	38	15	0	20	-	-
DPMP371	30	12	30	34	21	30	-	-
*P. brasiliense*								
DPMP55	11	10	34	18	16	26	-	-
DPMP224	30	12	36	27	15	26	-	-
DPMP372	31	14	36	26	28	32	-	-
DPMP374	34	16	37	27	24	34	-	-
DPMP396	18	14	40	32	18	27	-	-
DPMP120	0	12	30	30	26	30	+	-
DPMP135	10	10	24	22	14	26	-	-
DPMP152	10	0	24	26	13	27	-	-
*IFB5369	31	19	45	34	22	37	nt	nt
*P. carotovorum*								
DPMP189	10	8	13	0	11	30	-	-
DPMP199	0	7	8	5	2	7	+	-
DPMP200	8	7	12	10	11	5	-	-
DPMP323	35	17	35	26	24	34	-	-
DPMP399	30	15	38	27	20	29	-	-
*P. odoriferum*								
DPMP293	0	10	30	30	25	24	-	+
*P. parmentieri*								
DPMP136	0	11	30	27	20	20	-	+
*P. parvum*								
DPMP20	10	13	30	30	20	25	-	-
*IFB5220	30	13	37	32	24	28	nt	nt
*P. polaris*								
DPMP286	0	0	35	33	20	21	-	-
DPMP403	16	16	38	26	25	37	-	nt
*P. versatile*								
DPMP78	15	10	25	15	11	16	-	-
DPMP105	0	10	30	28	15	28	-	-
DPMP108	0	9	30	30	25	32	-	+
DPMP112	0	4	8	5	11	0	-	+
DPMP181	0	4	10	9	9	7	-	+
DPMP198	0	4	6	2	0	0	+	-
DPMP202	0	4	6	4	0	0	+	-
DPMP204	35	7	35	32	25	30	-	-
DPMP633	0	5	10	5	2	0	+	-
*P. zantedeschiae*								
* 9M^T^	8	0	15	29	18	25	-	-

* Strains isolated from plants with disease symptoms used as reference in this research. The symbol nt—means that strain was not tested; +/- strain was able to grow/did not grow on ESBL and CARBA chromagar plates.

## Data Availability

The data that support the findings of this study are available from the corresponding authors (K.W., M.W.), upon request. The nucleotide sequence data reported in this paper are available in the Genbank under the following accession numbers: ON381409-ON381476, MK024783, MK024784, KU510130, KU510135, KU510136, KU510109-KU510113, KU510119.

## Supplementary Materials

The following supporting information can be downloaded at: https://www.mdpi.com/article/10.3390/pathogens11070728/s1, Table S1: Results of pathogenicity tests. Table S2: Calculations of statistical significance; Figure S1 Comparison of the potato tissue maceration ability; Figure S2: Comparison of the chicory tissue maceration ability.

## References

[1] Lelliott R.A., Dickey R.S., Krieg N.R., Holt J.G. (1984). Genus VII. Erwinia. Bergey’s Manual of Systematic Bacteriology Williams & Wilkins, Baltimore.

[2] Mansfield J., Genin S., Magori S., Citovsky V., Sriariyanum M., Ronald P., Dow M., Verdier V., Beer S.V., Machado M.A. (2012). Top 10 Plant Pathogenic Bacteria in Molecular Plant Pathology. Mol. Plant Pathol..

[3] Toth I.K., Barny M., Brurberg M.B., Condemine G., Czajkowski R., Elphinstone J.G., Helias V., Johnson S.B., Moleleki L.N., Pirhonen M., Van Gijsegem F., van der Wolf J.M., Toth I.K. (2021). Pectobacterium and Dickeya: Environment to Disease Development. Plant Diseases Caused by Dickeya and Pectobacterium Species.

[4] Nabhan S., De Boer S.H., Maiss E., Wydra K. (2013). *Pectobacterium Aroidearum* sp. nov., a Soft Rot Pathogen with Preference for Monocotyledonous Plants. Int. J. Syst. Evol. Microbiol..

[5] Waleron M., Misztak A., Waleron M., Franczuk M., Jońca J., Wielgomas B., Mikiciński A., Popović T., Waleron K. (2019). *Pectobacterium zantedeschiae* sp. nov. a New Species of a Soft Rot Pathogen Isolated from Calla Lily (*Zantedeschia* spp.). Syst. Appl. Microbiol..

[6] Goto M., Matsumoto K. (1987). *Erwinia carotovora* subsp. *Wasabiae* subsp. nov. Isolated from Diseased Rhizomes and Fibrous Roots of Japanese Horseradish (Eutrema Wasabi Maxim.). Int. J. Syst. Bacteriol..

[7] Koh Y., Kim G., Lee Y., Sohn S., Koh H., Kwon S., Heu S., Jung J. (2012). *Pectobacterium carotovorum* subsp. *Actinidiae* subsp. nov., a New Bacterial Pathogen Causing Canker-like Symptoms in Yellow Kiwifruit, Actinidia Chinensis. N. Z. J. Crop Hortic. Sci..

[8] Gardan L., Gouy C., Christen R., Samson R. (2003). Elevation of Three Subspecies of Pectobacterium Carotovorum to Species Level: *Pectobacterium atrosepticum* sp. nov., *Pectobacterium betavasculorum* sp. nov. and *Pectobacterium wasabiae* sp. nov. Int. J. Syst. Evol. Microbiol..

[9] Alcorn S.M., Orum T.V., Steigerwalt A.G., Foster J.L.M., Fogleman J.C., Brenner D.J. (1991). Taxonomy and Pathogenicity of *Erwinia cacticida* sp. nov. Int. J. Syst. Bacteriol..

[10] Pérombelon M.C.M. (2002). Potato Diseases Caused by Soft Rot Erwinias: An Overview of Pathogenesis. Plant Pathol..

[11] Dees M.W., Lysøe E., Rossmann S., Perminow J., Brurberg M.B. (2017). *Pectobacterium polaris* sp. nov., Isolated from Potato (*Solanum tuberosum*). Int. J. Syst. Evol. Microbiol..

[12] Pasanen M., Waleron M., Schott T., Cleenwerck I., Misztak A., Waleron K., Pritchard L., Bakr R., Degefu Y., van der Wolf J. (2020). *Pectobacterium parvum* sp. nov., Having a Salmonella SPI-1-like Type III Secretion System and Low Virulence. Int. J. Syst. Evol. Microbiol..

[13] Khayi S., Cigna J., Chong T.M., Quêtu-Laurent A., Chan K.-G., Hélias V., Faure D. (2016). Transfer of the Potato Plant Isolates of Pectobacterium Wasabiae to *Pectobacterium parmentieri* sp. nov. Int. J. Syst. Evol. Microbiol..

[14] Waleron M., Misztak A., Waleron M., Franczuk M., Wielgomas B., Waleron K. (2018). Transfer of *Pectobacterium carotovorum* subsp. Carotovorum Strains Isolated from Potatoes Grown at High Altitudes to *Pectobacterium peruviense* sp. nov. Syst. Appl. Microbiol..

[15] Sarfraz S., Riaz K., Oulghazi S., Cigna J., Sahi S.T., Khan S.H., Faure D. (2018). *Pectobacterium punjabense* sp. nov., Isolated from Blackleg Symptoms of Potato Plants in Pakistan. Int. J. Syst. Evol. Microbiol..

[16] Cigna J., Laurent A., Waleron M., Waleron K., Dewaegeneire P., van der Wolf J., Andrivon D., Faure D., Hélias V. (2021). European Population of *Pectobacterium punjabense*: Genomic Diversity, Tuber Maceration Capacity and a Detection Tool for This Rarely Occurring Potato Pathogen. Microorganisms.

[17] Waleron M., Waleron K., Lojkowska E. (2014). Characterization of *Pectobacterium carotovorum* subsp. Odoriferum Causing Soft Rot of Stored Vegetables. Eur. J. Plant Pathol..

[18] Oulghazi S., Sarfraz S., Zaczek-Moczydłowska M.A., Khayi S., Ed-Dra A., Lekbach Y., Campbell K., Novungayo Moleleki L., O’Hanlon R., Faure D. (2021). Pectobacterium Brasiliense: Genomics, Host Range and Disease Management. Microorganisms.

[19] Portier P., Pédron J., Taghouti G., Fischer-Le Saux M., Caullireau E., Bertrand C., Laurent A., Chawki K., Oulgazi S., Moumni M. (2019). Elevation of *Pectobacterium carotovorum* subsp. Odoriferum to Species Level as *Pectobacterium odoriferum* sp. nov., Proposal of *Pectobacterium brasiliense* sp. nov. and *Pectobacterium actinidiae* sp. nov., Emended Description of Pectobacterium Carotovorum and Description of *Pectobacterium versatile* sp. nov., Isolated from Streams and Symptoms on Diverse Plants. Int. J. Syst. Evol. Microbiol..

[20] Welte C.U., de Graaf R.M., van den Bosch T.J.M., Op den Camp H.J.M., van Dam N.M., Jetten M.S.M. (2016). Plasmids from the Gut Microbiome of Cabbage Root Fly Larvae Encode SaxA That Catalyses the Conversion of the Plant Toxin 2-Phenylethyl Isothiocyanate. Environ. Microbiol..

[21] Jonkheer E.M., Brankovics B., Houwers I.M., van der Wolf J.M., Bonants P.J.M., Vreeburg R.A.M., Bollema R., de Haan J.R., Berke L., Smit S. (2021). The *Pectobacterium* Pangenome, with a Focus on *Pectobacterium brasiliense*, Shows a Robust Core and Extensive Exchange of Genes from a Shared Gene Pool. BMC Genom..

[22] Oulghazi S., Cigna J., Lau Y.Y., Moumni M., Chan K.G., Faure D. (2019). Transfer of the Waterfall Source Isolate *Pectobacterium carotovorum* M022 to *Pectobacterium fontis* sp. nov., a Deep-Branching Species within the Genus *Pectobacterium*. Int. J. Syst. Evol. Microbiol..

[23] Pédron J., Bertrand C., Taghouti G., Portier P., Barny M.-A. (2019). *Pectobacterium aquaticum* sp. nov., Isolated from Waterways. Int. J. Syst. Evol. Microbiol..

[24] Waleron M., Misztak A., Waleron M., Jonca J., Furmaniak M., Waleron K. (2019). *Pectobacterium polonicum* sp. nov. Isolated from Vegetable Fields. Int. J. Syst. Evol. Microbiol..

[25] Ben Moussa H., Pédron J., Bertrand C., Hecquet A., Barny M.-A. (2021). *Pectobacterium quasiaquaticum* sp. nov., Isolated from Waterways. Int. J. Syst. Evol. Microbiol..

[26] du Raan S., Coutinho T.A., van der Waals J.E. (2016). Cardinal Temperature Differences, Determined in Vitro, between Closely Related Species and Subspecies of Pectinolytic Bacteria Responsible for Blackleg and Soft Rot on Potatoes. Eur. J. Plant Pathol..

[27] Pasanen M., Laurila J., Brader G., Palva E.T., Ahola V., van Der Wolf J., Hannukkala A., Korhonen M. (2013). Characterisation of Pectobacterium Wasabi and *Pectobacterium carotovorum* subsp. Carotovorum Isolates from Diseased Potato Plants in Finland. Ann. Appl. Biol..

[28] van der Merwe J.J., Coutinho T.A., Korsten L., van der Waals J.E. (2010). *Pectobacterium carotovorum subsp*. Brasiliensis Causing Blackleg on Potatoes in South Africa. Eur. J. Plant Pathol..

[29] De Boer S.H. (1995). PCR Detection of *Erwinia carotovora* subsp. Atroseptica Associated with Potato Tissue. Phytopathology.

[30] Duarte V., De Boer S., Ward L., De Oliveira A.M.R. (2004). Characterization of Atypical Erwinia Carotovora Strains Causing Blackleg of Potato in Brazil. J. Appl. Microbiol..

[31] De Boer S.H., Li X., Ward L.J. (2012). *Pectobacterium* spp. Associated with Bacterial Stem Rot Syndrome of Potato in Canada. Phytopathology.

[32] Darrasse A., Priou S., Kotoujansky A., Bertheau Y. (1994). PCR and Restriction Fragment Length Polymorphism of a Pel Gene as a Tool to Identify Erwinia Carotovora in Relation to Potato Diseases. Appl. Environ. Microbiol..

[33] Nabhan S., De Boer S.H., Maiss E., Wydra K. (2012). Taxonomic Relatedness between *Pectobacterium carotovorum* subsp. *Carotovorum*, *Pectobacterium carotovorum* subsp. *Odoriferum* and *Pectobacterium carotovorum* subsp. *Brasiliense* subsp. nov. J. Appl. Microbiol..

[34] Versalovic J., Koeuth T., Lupski J.R. (1991). Distribution of Repetitive DNA Sequences in Eubacteria and Application to Fingerprinting of Bacterial Genomes. Nucleic Acids Res..

[35] Nassar A., Darrasse A., Lemattre M., Kotoujansky A., Dervin C., Vedel R., Bertheau Y. (1996). Characterization of Erwinia Chrysanthemi by Pectinolytic Isozyme Polymorphism and Restriction Fragment Length Polymorphism Analysis of PCR-Amplified Fragments of Pel Genes. Appl. Environ. Microbiol..

[36] Portier P., Pédron J., Taghouti G., Dutrieux C., Barny M.-A. (2020). Updated Taxonomy of *Pectobacterium* Genus in the CIRM-CFBP Bacterial Collection: When Newly Described Species Reveal “Old” Endemic Population. Microorganisms.

[37] Waleron M., Waleron K., Lojkowska E. (2001). Application of RecA PCR-RFLP for Differentiation of Erwinia Carotovora Associated with Potato Plants in Tuber. Plant Pathogenic Bacteria, Proceedings of the 10th International Conference on Plant Pathogenic Bacteria, Charlottetown, Prince Edward Island, Canada, 23–27 July 2000.

[38] Waleron M., Waleron K., Podhajska A.J., Łojkowska E. (2002). Genotyping of Bacteria Belonging to the Former Erwinia Genus by PCR-RFLP Analysis of a RecA Gene Fragment. Microbiology.

[39] Ben Moussa H., Bertrand C., Rochelle-Newall E., Fiorini S., Pédron J., Barny M.-A. (2022). The Diversity of Soft Rot Pectobacteriaceae along the Durance River Stream in the South-East of France Revealed by Multiple Seasonal Surveys. Phytopathology.

[40] Ashmawy N.A., Jadalla N.M., Shoeib A.A., El-Bebany A.F. (2015). Identification and Genetic Characterization of *Pectobacterium* spp. and Related Enterobacteriaceae Causing Potato Soft Rot Diseases in Egypt. J. Pure Appl. Microbiol..

[41] Oulghazi S., Moumni M., Khayi S., Robic K., Sarfraz S., Lopez-Roques C., Vandecasteele C., Faure D. (2020). Diversity of Pectobacteriaceae Species in Potato Growing Regions in Northern Morocco. Microorganisms.

[42] Zaczek-Moczydłowska M.A., Fleming C.C., Young G.K., Campbell K., O’Hanlon R. (2019). Pectobacterium and Dickeya Species Detected in Vegetables in Northern Ireland. Eur. J. Plant Pathol..

[43] Curland R.D., Mainello A., Perry K.L., Hao J., Charkowski A.O., Bull C.T., McNally R.R., Johnson S.B., Rosenzweig N., Secor G.A. (2021). Species of Dickeya and Pectobacterium Isolated during an Outbreak of Blackleg and Soft Rot of Potato in Northeastern and North Central United States. Microorganisms.

[44] Tsror (Lahkim) L., Hélias V., Mordechai-Lebiush S., Erlich O., Hazanovsky M., Chalupowicz L., Reuven M., Dror O., Valinsky L., Laurent A. (2021). Characterization of Pectobacterium Brasiliense Strains from Potato and Vegetables in Israel. Plant Pathol..

[45] Laurila J., Ahola V., Lehtinen A., Joutsjoki T., Hannukkala A., Rahkonen A., Pirhonen M. (2008). Characterization of Dickeya Strains Isolated from Potato and River Water Samples in Finland. Eur. J. Plant Pathol..

[46] Degefu Y. (2021). Co-Occurrence of Latent Dickeya and Pectobacterium Species in Potato Seed Tuber Samples from Northern Finland. Agric. Food Sci..

[47] Dees M.W., Lebecka R., Perminow J.I.S., Czajkowski R., Grupa A., Motyka A., Zoledowska S., Śliwka J., Lojkowska E., Brurberg M.B. (2017). Characterization of Dickeya and Pectobacterium Strains Obtained from Diseased Potato Plants in Different Climatic Conditions of Norway and Poland. Eur. J. Plant Pathol..

[48] Waleron M., Waleron K., Lojkowska E. (2015). First Report of *Pectobacterium carotovorum* subsp. Brasiliense Causing Soft Rot on Potato and Other Vegetables in Poland. Plant Dis..

[49] Waleron M., Waleron K., Lojkowska E. (2013). Occurrence of Pectobacterium Wasabiae in Potato Field Samples. Eur. J. Plant Pathol..

[50] Waleron M., Misztak A., Jońca J., Waleron K. (2019). First Report of Pectobacterium Polaris Causing Soft Rot of Potato in Poland. Plant Dis..

[51] Waleron M., Misztak A., Jońca J., Furmaniak M., Waleron M.M., Waleron K. (2019). First Report of ‘Candidatus Pectobacterium Maceratum’ Causing Soft Rot of Potato in Poland. Plant Dis..

[52] Del M., Marquez-Villavicencio P., Charkowski A. (2011). Soft Rot Disease Severity Is Affected by Potato Physiology and Pectobacterium Taxa. Plant Dis..

[53] Shyntum D.Y., Nkomo N.P., Shingange N.L., Gricia A.R., Bellieny-Rabelo D., Moleleki L.N. (2019). The Impact of Type VI Secretion System, Bacteriocins and Antibiotics on Bacterial Competition of *Pectobacterium carotovorum* subsp. Brasiliense and the Regulation of Carbapenem Biosynthesis by Iron and the Ferric-Uptake Regulator. Front. Microbiol..

[54] Niemi O., Laine P., Koskinen P., Pasanen M., Pennanen V., Harjunpää H., Nykyri J., Holm L., Paulin L., Auvinen P. (2017). Genome Sequence of the Model Plant Pathogen *Pectobacterium carotovorum* SCC1. Stand. Genom. Sci..

[55] Queenan A.M., Bush K. (2007). Carbapenemases: The Versatile β-Lactamases. Clin. Microbiol. Rev..

[56] Cohen M.L. (1992). Epidemiology of Drug Resistance: Implications for a Post-Antimicrobial Era. Science.

[57] Livermore D.M. (2009). Has the Era of Untreatable Infections Arrived?. J. Antimicrob. Chemother..

[58] Jonca J., Waleron M., Czaplewska P., Bogucka A., Steć A., Dziomba S., Jasiecki J., Rychłowski M., Waleron K. (2021). Membrane Vesicles of Pectobacterium as an Effective Protein Secretion System. Int. J. Mol. Sci..

[59] Royer G., Dixit Z., Pédron J., Pierrat G., Demontant V., Berçot B., Rodriguez C., Barny M.-A., Jacquier H., Woerther P.-L. (2022). Genetic and Phenotypic Study of the *Pectobacterium versatile* Beta-Lactamase, the Enzyme Most Similar to the Plasmid-Encoded TEM-1. Appl. Environ. Microbiol..

[60] Haas A.R.C. (1941). pH Determination in plant tissue. Plant Physiol..

[61] Masoero G., Cugnetto A. (2018). The Raw PH in Plants: A Multifaceted Parameter. J. Agron. Res..

[62] Hélias V., Hamon P., Huchet E., Wolf J.V.D., Andrivon D. (2012). Two New Effective Semiselective Crystal Violet Pectate Media for Isolation of Pectobacterium and Dickeya. Plant Pathol..

[63] Pinheiro J., Bates D., DebRoy S., Sarkar D., R Core Team (2021). NLME: Linear and Nonlinear Mixed Effects Models.

[64] William S., Feil H., Copeland A. (2012). Bacterial Genomic DNA Isolation Using CTAB.

[65] Kearse M., Moir R., Wilson A., Stones-Havas S., Cheung M., Sturrock S., Buxton S., Cooper A., Markowitz S., Duran C. (2012). Geneious Basic: An Integrated and Extendable Desktop Software Platform for the Organization and Analysis of Sequence Data. Bioinformatics.

