# Supplementary material for: The International Trade of Ware Vegetables and Orna-Mental Plants—An Underestimated Risk of Accelerated Spreading of Phytopathogenic Bacteria in the Era of Globalisation and Ongoing Climatic Changes
Source: Pathogens. 2022 Jun 26;11(7):728. doi: 10.3390/pathogens11070728 (PMC9319320; doi:10.3390/pathogens11070728)
Supplement: Supplementary file 1 [file pathogens-11-00728-s001.zip › pathogens-1719330-supplementary.pdf]

Table S1. Results of pathogenicity tests.

Strain	Host	Mean diameter of rotting potato tissue ± Standard Deviation [mm]	Mean surface area of rotting chicory tissue ± Standard Deviation [cm^2]
Pectobacterium atrosepticum			
DPMP134	Potato tuber	20.7 ± 1.5	17.2 ± 1.7
DPMP634	Potato tuber	15.7 ± 4.5	0.9 ± 0.5
***IFB5050**	Potato stem	20.2 ± 1.0	27.1±1.2
***IFB5205**	Potato stem	24.0 ± 1.7	nt
***ICMP1526^T**	Potato stem	8.6 ± 1.3	0
Pectobacterium brasiliense			
DPMP224	Potato tuber	12.3 ± 1.6	11.2 ± 1.3
DPMP374	Potato tuber	21.3 ± 0.6	16.2 ± 0.6
DPMP396	Bittersweet	15.7 ± 1.0	38.1 ± 0.9
DPMP55	Potato tuber	21.0 ± 4.6	14.1 ± 1.1
***IFB5258**	Sugar Beet	15.7 ± 1.0	2.1 ± 0.1
***IFB5369**	Potato tuber	21.0 ± 2.0	16.91 ± 0.9
*** LMG2137^T**	Potato tuber	13.0 ± 1.0	10.6 ± 0.4
Pectobacterium carotovorum			
DPMP189	Potato tuber	19.7 ± 1.6	13.2 ± 0.4
DPMP199	Potato tuber	14.0 ± 2.6	17.4 ± 0.7
DPMP200	Potato tuber	17.9 ± 1.0	37.7 ± 0.9
DPMP399	Sugar beet	22.0 ± 0.9	23.1 ± 0.6
***DPMP510**	Potato stem	13.0 ± 0.9	11.4 ± 0.6
***LMG2401**	Carrot	6.0 ± 1.0	nt
***LMG2404^T**	Potato	12.6 ± 0.9	0
Pectobacterium parmentieri			
DPMP136	Potato tuber	15.7 ± 0.7	25.7 ±1.9
***IFB5322**	Potato stem	17..6 ± 0.4	0
***SCC3193**	Potato	30.0 ± 1.2	1.9 ± 0.1
Pectobacterium parvum			
DPMP20	Potato	23.4 ± 0.5	38.5 ± 1.6
***IFB5220**	Potato stem	15.4 ± 2.4	39.5 ± 3.5
***s421^T**	Potato	14.3 ± 2.3	nt
Pectobacterium odoriferum			
DPMP293	Celery	19.2 ± 4.0	13.7 ± 1.1
***IFB5295**	Carrot	16.2 ± 0.4	11.7 ± 2.0
***CFBP1878^T**	Chicory	11.6 ± 0.8	9.5 ± 1.8
Pectobacterium polaris			
DPMP286	Potato	19.2 ± 4.7	36.2 ± 3.7
DPMP380	Sugar beet	12.5 ± 2.5	26.8 ± 1.9
DPMP397	Bittersweet	8.7 ± 0.6	8.6 ± 1.0
DPMP403	Sugar beet	22.2 ± 2.4	12.4 ± 1.8
***IFB5222**	Potato stem	10.6± 1.0	36.2 ± 5.8
***NIBIO1006^T**		12.5 ± 4.4	42.5 ± 4.5
Pectobacterium versatile			
DPMP204	Potato	22.0 ± 4.0	21.8 ± 2.9
DPMP387	Red beet	16.3 ± 4.7	29.7 ± 3.1
DPMP335	Peppers felepano	25.5 ± 3.0	24.7 ± 3.9
DPMP248	Potato	18.9 ± 2.3	16.5 ± 2.2
DPMP337	Fennel	19.3 ± 2.4	13.1 ± 2.9
DPMP198	Cactus	22.1 ± 2.4	17.8 ± 2.4
DPMP633	Potato tuber	19.3 ± 2.7	6.9 ± 1.0
***IFB5169**	Potato stem	35.4 ± 3.0	0
***SCC1**		14.0 ± 1.0	21.8 ± 3.4
Pectobacterium zantedeschiae			
**9M^T = DPMP423**	Calla lilly	21.9 ± 1.2	24.6 ± 10.4
Dickeya dadantii			
DPMP625		21.6 ± 1.3	0
*** 3937 ^{T}**		25.0 ± 5.0	13.06 ± 2.4
Negative control		0	0

* reference strains and strains that were isolated from plants with disease symptoms
^{T} – Type Strain

Table S2. Comparison of the potato tissue maceration ability of the 27 strains isolated from asymptomatic plant samples in contrast to 17 strains originating from plants with disease symptoms. Strains abbreviations: * strains isolated from symptomatic plants, ^{T} - type strains. Means ± SD of diameters of the rotten tissues is depicted. Three independent experiments with nine technical replications were conducted. Means marked with a different letter (a, b, c, … p) are significantly different according to the Games-Howell test followed by a post-hoc analysis applying Welch and Brown-Forsythe corrections for nonhomogeneous variances criterion at p < 0.02.

	x
* CFBP1878T	abcdef
* DPMP510	abcdegh
* ICMP1526 T	acfg
* IFB5050	ijklmno
* IFB5169	abcdefghijklmnop
* IFB5205	ijklmp
* IFB5222	abcdf
* IFB5258	cdeghijmno
* IFB5295	cdeghijkn
* IFB5322	ghijkn
* IFB5369	bdehijklmnop
* LMG2137 T	abcdegh
* LMG2404	abcdegh
* s421T	abcdefghijklmno
* SCC3193	p
*LMG2401	f
*NIBIO1006T	abcdefghijklmnop
*SCC1	abcdeghjn
9MT	iklm
DPMP134	ehijklmno
DPMP136	cdeghijn
DPMP189	ehijklmno
DPMP198	abcdeghijklmnop
DPMP199	abcdefghijklmnop
DPMP20	lp
DPMP200	hijklmno
DPMP204	abcdefghijklmnop
DPMP224	abcdefghno
DPMP248	abcdefghijklmnop
DPMP286	abcdefghijklmnop
DPMP293	abcdefghijklmnop
DPMP335	abcdefghijklmnop
DPMP337	abcdefghijklmnop
DPMP353	ijklmno
DPMP374	lmo
DPMP380	abcdefghijklmno
DPMP387	abcdefghijklmnop

DPMP396	cdeghijkn
DPMP397	abf
DPMP399	kl
DPMP403	abcdeghijklmnop
DPMP511	abcdefghijklmnop
DPMP55	abcdefghijklmnop
DPMP625	ijklm
DPMP633	abcdefghijklmnop
DPMP634	abcdefghijklmnop
* 3937 T	abcdefghijklmnop

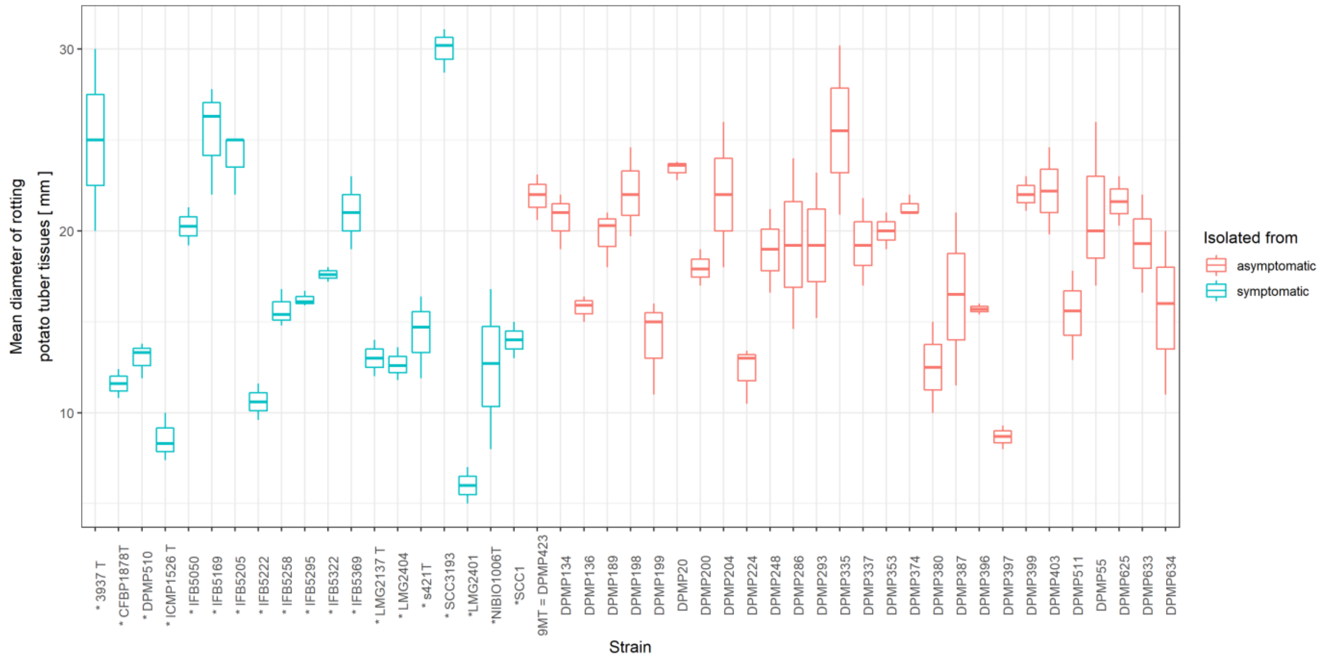

Figure S1. Comparison of the potato tissue maceration ability of the 26 strains isolated from asymptomatic plant samples in contrast to 21 strains originating from plants with disease symptoms. Strains abbreviations: * strains isolated from symptomatic plants, ^{T} - type strains.

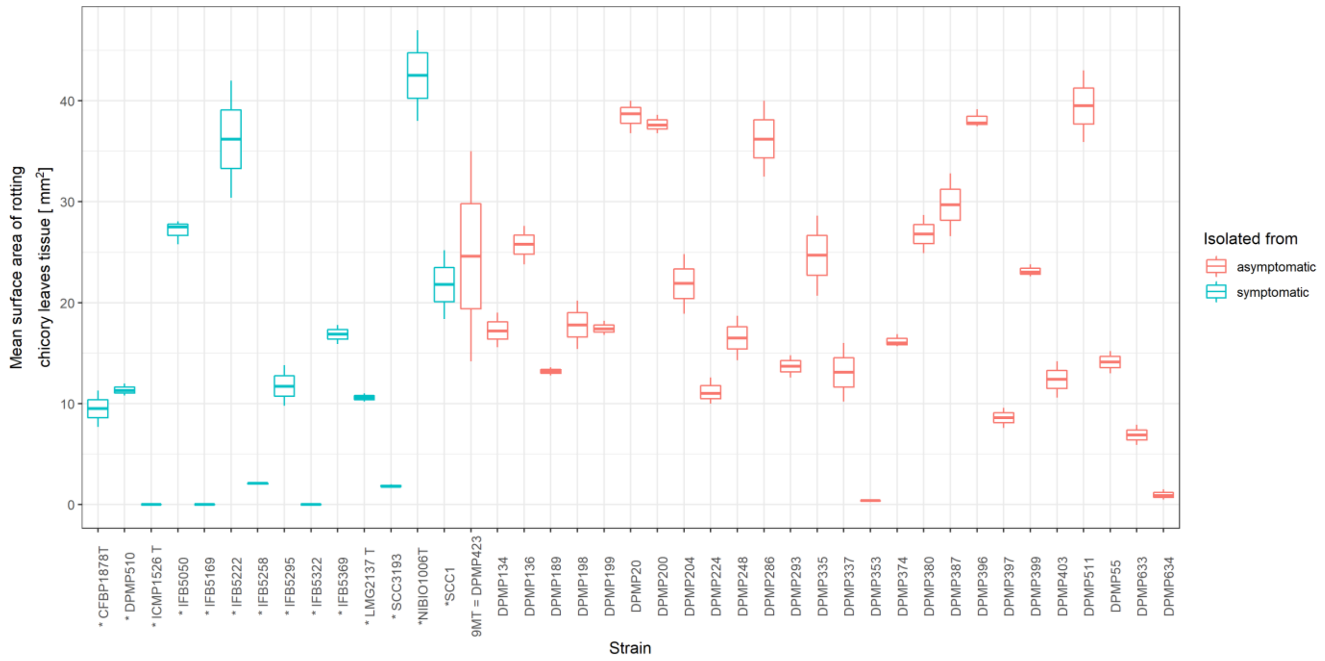

Figure S2. Comparison of the chicory tissue maceration ability of the 26 strains isolated from asymptomatic plant samples in contrast to 18 strains originating from plants with disease symptoms. Strains abbreviations: * strains isolated from symptomatic plants, T - type strains.
